# Effect of Dl-3-n-butylphthalide on mitochondrial Cox7c in models of cerebral ischemia/reperfusion injury

**DOI:** 10.3389/fphar.2023.1084564

**Published:** 2023-02-22

**Authors:** Jingjing Jia, Jianwen Deng, Haiqiang Jin, Jie Yang, Ding Nan, Zemou Yu, Weiwei Yu, Zhiyuan Shen, Yuxuan Lu, Ran Liu, Zhaoxia Wang, Xiaozhong Qu, Dong Qiu, Zhenzhong Yang, Yining Huang

**Affiliations:** ^1^ Department of Neurology, Peking University First Hospital, Beijing, China; ^2^ National Center for Children’s Health, Department of Neurology, Beijing Children’s Hospital, Capital Medical University, Beijing, China; ^3^ Leewe Biopharmaceutical Co., Ltd, Xianlin University, Nanjing, China; ^4^ Department of Hyperbaric Oxygen, Beijing Chaoyang Hospital, Capital Medical University, Beijing, China; ^5^ Department of Neurology, Peking University Shenzhen Hospital, Shenzhen, Guangdong, China; ^6^ Center of Materials Science and Optoelectronics Engineering, College of Materials Science and Opto-Electronic Technology, University of Chinese Academy of Sciences, Beijing, China; ^7^ Beijing National Laboratory for Molecular Sciences (BNLMS), Laboratory of Polymer Physics and Chemistry, CAS Research/Education Center for Excellence in Molecular Sciences, Institute of Chemistry, Chinese Academy of Sciences, Beijing, China; ^8^ Institute of Polymer Science and Engineering, Department of Chemical Engineering, Tsinghua University, Beijing, China

**Keywords:** cerebral ischemia/reperfusion, Dl-3-n-butylphthalide, cytochrome c oxidase, mitochondrial dysfunction, ROS

## Abstract

Several studies have demonstrated the protective effect of dl-3-n-Butylphthalide (NBP) against cerebral ischemia, which may be related to the attenuation of mitochondrial dysfunction. However, the specific mechanism and targets of NBP in cerebral ischemia/reperfusion remains unclear. In this study, we used a chemical proteomics approach to search for targets of NBP and identified cytochrome C oxidase 7c (Cox7c) as a key interacting target of NBP. Our findings indicated that NBP inhibits mitochondrial apoptosis and reactive oxygen species (ROS) release and increases ATP production through upregulation of Cox7c. Subsequently, mitochondrial respiratory capacity was improved and the HIF-1α/VEGF pathway was upregulated, which contributed to the maintenance of mitochondrial membrane potential and blood brain barrier integrity and promoting angiogenesis. Therefore, our findings provided a novel insight into the mechanisms underlying the neuroprotective effects of NBP, and also proposed for the first time that Cox7c exerts a critical role by protecting mitochondrial function.

## Introduction

Ischemic stroke is one of the leading causes of adult death and disability worldwide, placing a heavy burden on families and communities. Although thrombolytic therapy using recombinant tissue plasminogen activator (rt-PA) is the preferred treatment for acute ischemic stroke, strict time windows and exclusion criteria allow only 3–5% of patients to benefit (Adeoye et al., 2011). In addition, rt-PA treatment is associated with certain side effects, for example, it amplifies the neurotoxicity induced by hemoglobin and causes damage to neuronal cells (Wang et al., 2021). Furthermore, cerebral ischemia/reperfusion causes a series of biochemical cascades, which further worsens damage to brain tissue (i.e., ischemia/reperfusion injury) and weakens the beneficial effects of vascular recanalisation (Yang and Betz 1994; Aronowski et al., 1997).

Mitochondria play an important role in the pathological process of ischemia/reperfusion injury, and mitochondrial dysfunction is considered a marker of nerve cell death during ischemic stroke (Valko et al., 2007). Mitochondria are key regulators of cell survival during disease, contributing to cell survival by promoting ATP production and, conversely, inducing apoptosis by releasing pro-apoptotic factors (Anzell et al., 2018). When mitochondria are damaged, they produce a large number of reactive oxygen species (ROS) and induce oxidative stress reactions. During this process, cytochrome C (cytC) is released from mitochondria into the cytoplasm, and pro-apoptotic factors are upregulated, thereby inducing caspase cascades and triggering cell apoptosis (Krause et al., 1988; Sanderson et al., 2013).

Dl-3-n-butylphthalide (NBP) is a racemic compound, a yellow oily liquid with a celery-like aroma. Several studies have demonstrated the protective effect of NBP against cerebral ischemia, and the compound was approved by the China Food and Drug Administration (CFDA) for the treatment of acute ischemic stroke in 2002 (Huang et al., 2018). Research has indicated that NBP reduces the extent of the cerebral infarction area, alleviates cerebral oedema, and improves cerebral energy metabolism during cerebral ischemia (Chang and Wang 2003). In addition, the safety and effectiveness of NBP have been further verified in multiple clinical trials (Huang et al., 2018). The potential mechanisms underlying the neuroprotective effects of NBP include reducing oxidative stress, protecting mitochondrial function, reducing inflammatory responses, and reducing neuronal apoptosis (Xu et al., 2017; Zhao et al., 2017). Some studies have also shown that NBP can significantly increase ATP levels after ischemic injury (Ye et al., 2015), improve mitochondrial morphology, and maintain mitochondrial membrane potential (Li et al., 2010; Yin et al., 2016; Chen et al., 2018). Although these studies have explored the possible mechanisms of NBP and conducted certain analyses and hypothesis, it is not clear whether the effects of NBP on these molecules and pathways are direct or indirect, or whether they are caused by influencing other factors. Hence, the specific target and mechanism underlying the protective effects of NBP in mitochondria remain to be elucidated.

Therefore, in the present study, we aimed to conduct a comprehensive analysis of NBP and protein interactions using a quantitative chemical proteomics approach. This approach identified cytochrome C oxidase 7c (Cox7c) as one of the major targets of NBP. Our results further indicated that NBP can directly activate Cox7c in the mitochondria of vascular endothelial cells, increase the production of ATP, reduce the release of ROS, contribute to the stability of mitochondrial membrane potential, and maintain the integrity of the blood brain barrier. Our analysis also revealed that the knockdown of Cox7c also abolishes the beneficial effects of NBP. Thus, not only do our findings verify the positive effect of NBP on ischemic stroke, but they also provide a theoretical basis for subsequent studies regarding the role of mitochondria in cerebral ischemia/reperfusion injury and the use of NBP in the treatment of patients with ischemic stroke.

## Materials and methods

### Animals

In all experiments, we used male C57BL/6 mice (25–30 g), which were purchased from SPF Biotechnology Co., Ltd. (Beijing, China). All animal experiments were approved by the appropriate animal ethics committee. All experiments were conducted in accordance with the National Institutes of Health (NIH; Bethesda, MD, United States) Guide for the Care and Use of Laboratory Animals. The mice were raised under standard light (12-h light/dark cycle), temperature (20–25°C), and humidity (40–60%) conditions and were provided with free access to food and water. The mice were randomly assigned to individual groups.

### Middle cerebral artery occlusion/reperfusion (MCAO/R)

As described previously, the cerebral ischemia/reperfusion model was developed by inducing reperfusion 1.5 h after the occlusion of the right middle cerebral artery (de la Rosa et al., 2014). Mice were first anaesthetized *via* an intraperitoneal injection of 0.5% pentobarbital sodium. The right common carotid artery (CCA), external carotid artery (ECA), and internal carotid artery (ICA) were separated through a solemn incision in the neck. A small ECA incision was made, and a nylon filament with a diameter of approximately 0.22 mm was inserted through the incision, extending roughly 9–11 mm into the ICA. After 1.5 h, the nylon filaments were removed for reperfusion. Sham mice underwent the same procedure, but no filaments were inserted. From the beginning of the operation to the end of anaesthesia, each mouse’s body temperature was maintained at approximately 37°C by placing them on a thermostatic heating pad. We excluded mice that died during surgery and that failed to model MCAO/R.

### Vector transduction in mouse brain and bEnd.3 cells

For oeCox7c (overexpression of Cox7c) in mice, Adeno-associated virus(AAV) vectors encoding Cox7c with GFP and control vectors without Cox7c were customized from ViGene Biosciences (Jinan, China). Following previously established methods, a four-point injection was performed at the following coordinates: 0.5 mm anterior to the bregma, 2.0 or 3.0 mm lateral to the sagittal suture, and 1.0 or 2.8 mm from the surface of the skull. A total of 1 μL of concentrated AAV vectors was injected into each position at a rate of 0.5 μL/min with a 30-gauge needle on a 10-μL Hamilton syringe (catalog no. 80010) (Zeng et al., 2014). After the injection, the needle remained in position for 5 min before it was withdrawn. The animals were allowed to recover the wound for 14 days before being operated MCAO/R surgery.

For oeCox7c (overexpression of Cox7c) in bEnd.3 cells, lentiviral vectors encoding Cox7c with GFP and control vectors without Cox7c were customized from JTS scientific (Wuhan, China). The bEnd.3 cells were transinfected by lentiviral vectors as previously described (Huang et al., 2013). The cells were infected with the medium containing lentiviral vectors for 6 h and replaced with fresh medium for another 48 h. The green fluorescent protein signal was detected by a fluorescence microscope, then the cells were exposed to OGD/R.

### Cell cultures

To simulate ischemic conditions, the murine endothelial cell line bEnd.3 (purchased from the ATCC) was subjected to oxygen and glucose deprivation and reoxygenation (OGD/R) as an *in vitro* ischemia/reperfusion model. The bEnd.3 cells were seeded into 6-well plates and cultured in high-glucose DMEM (Gibco, China) with 10% fetal bovine serum and 1% penicillin-streptomycin. They were then cultured for 24 h in an incubator containing 5% CO_2_ at 37 °C. For cells subjected to OGD, the medium was replaced with glucose-free DMEM (Gibco, China), and the cells were placed in an anaerobic chamber containing 95% N_2_ and 5% CO_2_ at 37°C for 6 h. The cells were then returned to normal medium and incubated under normal conditions for 18 h to achieve reperfusion.

### Drug administration

Synthetic NBP was generously supplied by CSPC NBP Pharmaceutical Co., Ltd. (Shijiazhuang, China). The probe was synthesized and provided by WuXi AppTec Co., Ltd. (Wuxi, China). As previously reported, NBP was dissolved in corn oil and administered to MCAO mice at a dose of 60 mg/kg/day *via* oral gavage immediately after reperfusion, and mice were subjected to continuous administration for 3 days (Wang et al., 2021; Yang et al., 2019). Mice that received an equal volume of corn oil orally served as controls. On day 3 after MCAO/R, mouse brain tissues were harvested and used to perform subsequent experiments.

In accordance with a previously described protocol, NBP or probe was dissolved in DMSO, and 100 μM NBP or probe was added to the medium immediately after re-oxygenation of bEnd.3 cells, with an equal volume of DMSO for controls (Yang et al., 2019). In the NBP + probe group, cells were co-treated with 100 µM NBP and 100 µM probe. On 18 h after OGD/R, bEnd.3 cells were harvested and used to perform subsequent experiments.

### Neurological function assessment

As previously reported, on day 3 after MCAO/R, the mNSS (Modified Neurological Severity Score) was used to evaluate neurological deficits (Chen et al., 2001). The mNSS score ranges from 0 to 18. Scores ranging from 13 to 18 indicate serious injury, those ranging from 7–12 indicate moderate injury, and those ranging from one to six indicate minor injury. The mNSS evaluations were performed by investigators who were blinded to the treatment groups. Six mice were used in each group.

### 2,3,5-Triphenyltetrazolium chloride (TTC) staining

Infarct volume was assessed *via* TTC (Sigma-Aldrich, United States) staining. Six mice were used in each group in this experiment. The mice were anaesthetized, following which their brains were perfused with normal saline, removed, and immediately frozen at -20°C for 20 min. Brains were cut into sections of approximately 1 mm, and the sections were immersed in 2% TTC solution at 37°C for 20 min. The infarct volume was determined by measuring the unstained area using the image analysis software ImageJ (Version 1.53) (Liu et al., 2017).

### Determination of blood brain barrier permeability

Five mice were used in each group in this experiment. Following injection of 2% EB (Evans Blue) into the tail vein of mice at 2 ml/kg for 2 h, mice were sacrificed, and their brains were collected and weighed after perfusion with normal saline. The brain tissue was placed in 5 ml of formamide and bathed at 60 °C for 72 h. Then, the optical density was measured at a wavelength of 620 nm using a microplate reader (BioTek, United States). Standard curves were drawn to distribute EB content in brain tissue. EB content was determined using the following formula: EB content in brain tissue (μg/mg wet brain) = EB concentration (μg/ml) x formamide (ml)/wet weight (mg).

### Pulldown experiment and click-reaction-assisted activity-based protein profiling (ABPP)

The pulldown experiment is an *in vitro* technique used to detect physical interactions between two or more proteins and is a valuable tool for confirming predicted protein-protein interactions or identifying new interaction partners. ABPP is a powerful method that can help to identify the cellular targets of bioactive molecules. In general, the probe molecule is designed to insert a terminal acetylene into the bioactive parent molecule to facilitate Cu(I)-catalysed click reactions with azide affinity markers (Darabedian et al., 2018; Li et al., 2019). The probe used in our experiment is a molecule formed by inserting a terminal acetylene into the molecule of NBP, so it can pulldown the binding protein *via* ABPP. We dissolved NBP or probe in DMSO and added 100 μM of NBP or probe to the medium of treated bEnd.3 cells immediately after reoxidation. The treated cells were collected 18 h after reoxygenation and placed in RIPA lysis buffer (Beyotime, China) to extract the proteins. The protein solution was incubated at 20–25 °C for 1 h with 100 μM Biotin azide (Sigma-Aldrich, United States), 1.0 mM CuSO4 (Sigma-Aldrich, United States), 100 μM THTPA (Sigma-Aldrich, United States), and 100 μM NaVc (Sigma-Aldrich, United States) for the “click” reaction. Subsequently, 900 μL of buffer (50 mM Tris-HCl, pH 7.4, 0.15 M NaCl, 0.1% SDS) and 40 μL of streptavidin-sepharose beads (Sigma-Aldrich, United States) were added, rotating continuously overnight at 4°C. After washing the beads with buffer three times, the eluted protein was separated *via* SDS-PAGE. Afterward, the eluted protein was stained using a Fast Silver Stain Kit (Beyotime, China) or transferred to nitrocellulose membranes (Millipore, United States) for Western blot analysis.

### Mass spectrometry analysis

The proteins were extracted from the treated bEnd.3 cells, and the resulting peptides were separated *via* capillary liquid chromatography/tandem mass spectrometry (LC-MS/MS). All analyses were performed using a Q-Exactive mass spectrometer (Thermo, United States) equipped with a Nanospray Flex source (Thermo, United States). The peptides were identified using the National Center for Biotechnology Information (NCBI) protein database.

### RNA interference

Lipofectamine 2000 (Invitrogen, United States) was used to interfere with bEnd.3 cells before OGD/R treatment. The bEnd.3 cells were seeded into 6-well plates (50–70% confluent) and transfected with 100 pmol of appropriate siRNA duplex in media and incubated for 6 h. The cells were placed in new culture medium for 48 h, following which OGD/R was performed. The siRNA sequences used were shown as follows: Ndufa11-F: 5′- GAU​GUU​CCA​ACU​UUG​AAT​T-3′, Ndufa11-R:5′- UUC​AAA​GUU​GGA​ACA​CAU​CTT -3′; Ndufb5-F: 5′- GGA​AGU​UGU​GAA​UUC​CUA​ATT -3′, Ndufb5-R:5′- UUA​GGA​AUU​CAC​AAC​UUC​CTT -3′; Cox3-F: 5′- CCA​AUA​UCC​UCA​CAA​UAU​ATT -3′, Cox3-R:5′- UAU​AUU​GUG​AGG​AUA​UUG​GTT -3′; Cox7c -F: 5′- GAU​GAC​CGU​GUA​CUU​UGG​AUC​UGG​A -3′, Cox7c -R:5′- UCC​AGA​UCC​AAA​GUA​CAC​GGU​CAU​C -3′.

### Measurement of mitochondrial respiration in bEnd.3 cells

As previously described, the high-resolution Oroboros Oxygraph 2K (Oroboros Instruments, Innsbruck, Austria) was used to assess mitochondrial respiration rates in bEnd.3 cells using two 2-ml chambers under continuous stirring at 37°C (Abdel-Rahman et al., 2016; Roy Chowdhury et al., 2020). The treated bEnd.3 cells were washed with PBS and then suspended in DMEM medium and transferred into the O2k chambers. At the beginning of the experiment, the oxygen flux is monitored for at least 10 min to determine routine respiration. Oligomycin (Omy) is injected through the stoppers to detect LEAK respiration, which indicates that ATP synthase is inhibited and protons leak across the inner mitochondrial membrane. Next, Carbonyl cyanide m-chlorophenyl hydrazone (CCCP) is sequentially injected into the chambers until the respiratory flux reaches a plateau to determine the maximum capacity of the electronic transmission system. The difference in values between maximum respiration and Routine respiration represents the spare respiratory capacity for mitochondrial ATP production (Roy Chowdhury et al., 2020). The respiratory control ratio is defined as the ratio of uncoupled respiration rates to the LEAK respiratory rates, and it represents the mitochondrial respiration capacity (Hall et al., 2013; Roy Chowdhury et al., 2020). Data acquisition and graphic presentation were performed with the DatLab® software, version 4.3 (Oroboros Instruments).

### Cell viability assay

Cell viability was estimated using the Cell Counting Kit 8 (CCK8, Dojindo, Japan). The cells were cultured in 96-well plates, and 10 μL CCK8 reagent was added to each well. They were then incubated at 37°C for 2–3 h, and the absorbance was measured at 450 nm to calculate cell viability.

### Real-time PCR

Total RNA was extracted from brain tissue and bEnd.3 cells using Trizol reagent (Invitrogen, United States). Next, 1 µg mRNA was reverse transcribed into cDNA using the PrimeScriptTM RT kit (TransGen, China). SYBR Green PCR Master Mix (Applied Biosystems, United States) was used for real-time quantitative PCR on an ABI 7500 FAST real-time PCR System (Applied Biosystems, United States). The primers were listed as follows:Cox7c-F:5′-ATGTTGGGCCAGAGTATCCG-3′,Cox7c-R:5′-ACCCAGATCCAAAGTACACGG-3′; VEGFA-F:5′-GCACATAGAGAGAATGAGCTTCC-3′, VEGFA-R: 5′- CTC​CGC​TCT​GAA​CAA​GGC​T-3′; VEGFB-F: 5′- GCC​AGA​CAG​GGT​TGC​CAT​AC -3′, VEGFB-R:5′-GGAGTGGGATGGATGATGTCAG -3′; β-actin-F: 5′- GGC​TGT​ATT​CCC​CTC​CAT​CG-3′, β-actin-R: 5′- CCA​GTT​GGT​AAC​AAT​GCC​ATG​T-3′. Target gene expression levels were determined relative to the β-actin expression level using the ΔΔCT method.

### Western blot analysis

Three mice were used in each group in this experiment. Tissues of the cerebral hemisphere on the infarct side or cells were lysed in RIPA lysis buffer (Beyotime, China) containing 1 mM of phenylmethylsulfonyl fluoride (PMSF, Beyotime, China). For the extraction of mitochondria and cytoplasmic proteins, all steps were performed in accordance with the instructions of the Tissue Mitochondria Isolation Kit (Beyotime, China) and the Cell Mitochondria Isolation Kit (Beyotime, China). The protein concentration was determined using the BCA kit (Beyotime, China). The pyrolysis materials were separated *via* SDS-PAGE and transferred onto nitrocellulose membranes (Millipore, United States). The membranes were blocked with TBST containing 5% skim milk for 1 h, following which they were incubated with primary antibody overnight at 4°C. Primary antibodies used included the following: Cox7c (1:1000, 11411, Proteintech), VDAC1 (1:1000, 55259, Proteintech), Occludin (1:1000, 13409, Proteintech), ZO-1 (1:1000, 21773, Proteintech), HIF-1α (1:1000, 36169, CST), β-actin (1:1000, 3700, CST). The membranes were washed with TBST three times and incubated with the corresponding secondary antibody at room temperature for 1 h. After washing three times, the electrochemiluminescence (ECL) detection system (Syngene, England) was used for development. ImageJ software was used to quantitatively analyse the protein bands, and the results were normalized to β-actin levels.

### Immunofluorescence staining

Harvested brains were fixed in 4% paraformaldehyde and prepared into 10-mm frozen sections, and three mice were used in each group. The bEnd.3 cells were fixed on the slides with 4% paraformaldehyde. The samples were blocked with 10% goat serum at room temperature for 1 h, following which they were incubated overnight at 4°C with an appropriate primary antibody. The samples were then incubated with the corresponding secondary antibody at room temperature for 1 h and mounted with a medium containing DAPI (Zsbio, China). The following primary and secondary antibodies were used: Cox7c (1:100, 11411, Proteintech), CD31 (1:100, ab24590, Abcam), Alexa Fluor 488 (1:200, A32731, Invitrogen), Alexa Fluor 594 (1:200, A32744, Invitrogen). The detection of cell apoptosis and mitochondrial localization were performed according to the instructions of the One Step TUNEL Apoptosis Assay Kit (Beyotime, China) and Mito-Tracker Red CMXRos (Beyotime, China). A fluorescence microscope (200×; Eclipse 90i, Nikon, Japan) was used to acquire images. The immunofluorescence staining images of brains were from the cortex on the infarct side.

To assess hypoxia levels, three mice were used in each group in this experiment. Mice were injected with a 60 mg/kg hypoxyprobe (Burlington, United States) into the tail vein, following which mice were sacrificed and their brains were removed 1.5 h later. Tissue sections were incubated overnight at 4°C with anti-hypoxyprobe (1:100, FITC-conjugated mouse immunoglobulin G (IgG) monoclonal antibody). After washing, all sections were mounted on slides with a medium containing DAPI. The images were acquired using a fluorescence microscope (40×; Eclipse 90i, Nikon, Japan).

### Flow cytometry

Mitochondrial membrane potential was examined using the MitoProbe™ JC-1 Assay Kit for Flow Cytometry (Invitrogen, United States). The cells were incubated at 37°C with 5% CO_2_ in a medium containing 2 μM JC-1 for 30 min. The cells were resuspended in phosphate buffered saline (PBS), and the fluorescence emission shift from green (∼529 nm) to red (∼590 nm) was detected *via* flow cytometry (BD, United States). The red/green fluorescence ratio indicates mitochondrial membrane potential. ROS levels were measured *via* DHE (Keygene, China) staining. The cells were incubated in a medium containing 5 μM DHE at 37°C for 30 min in the dark. The cells were resuspended in PBS, and the fluorescence intensity at 620 nm was measured using a flow cytometer. FlowJo software (Tree Star Inc., United States) was used for data analysis.

### ATP detection

ATP levels in brain tissues and cells were detected using an ATP Assay Kit (Beyotime, China). Three mice were used in each group in this experiment. The protein was extracted from brain tissue and bEnd.3 cells, and the protein concentration was detected using a BCA kit. After mixing 20 μL of protein solution with 100 μL of ATP detection reagent, luminance (relative light units (RLUs)) was measured using a multifunctional microplate (BioTek, United States). The concentration of ATP was calculated according to the standard ATP curve.

### Statistical analysis

GraphPad Prism 8 (GraphPad Software Inc., United States) was used for statistical analysis. The student’s t-test was used to determine the significance of differences between the two groups. When the difference between the groups was statistically significant, one-way analyses of variance (ANOVA) were performed, followed by Tukey’s *post hoc* test. Two-way ANOVA accompanied by a Bonferroni *post hoc* test was performed for multiple comparisons. The data are presented as the mean ± standard error of the mean (SEM). *p* < 0.05 was considered statistically significant.

## Results

### NBP exerts neuroprotective effects in cerebral ischemia/reperfusion mice

**FIGURE 1:**
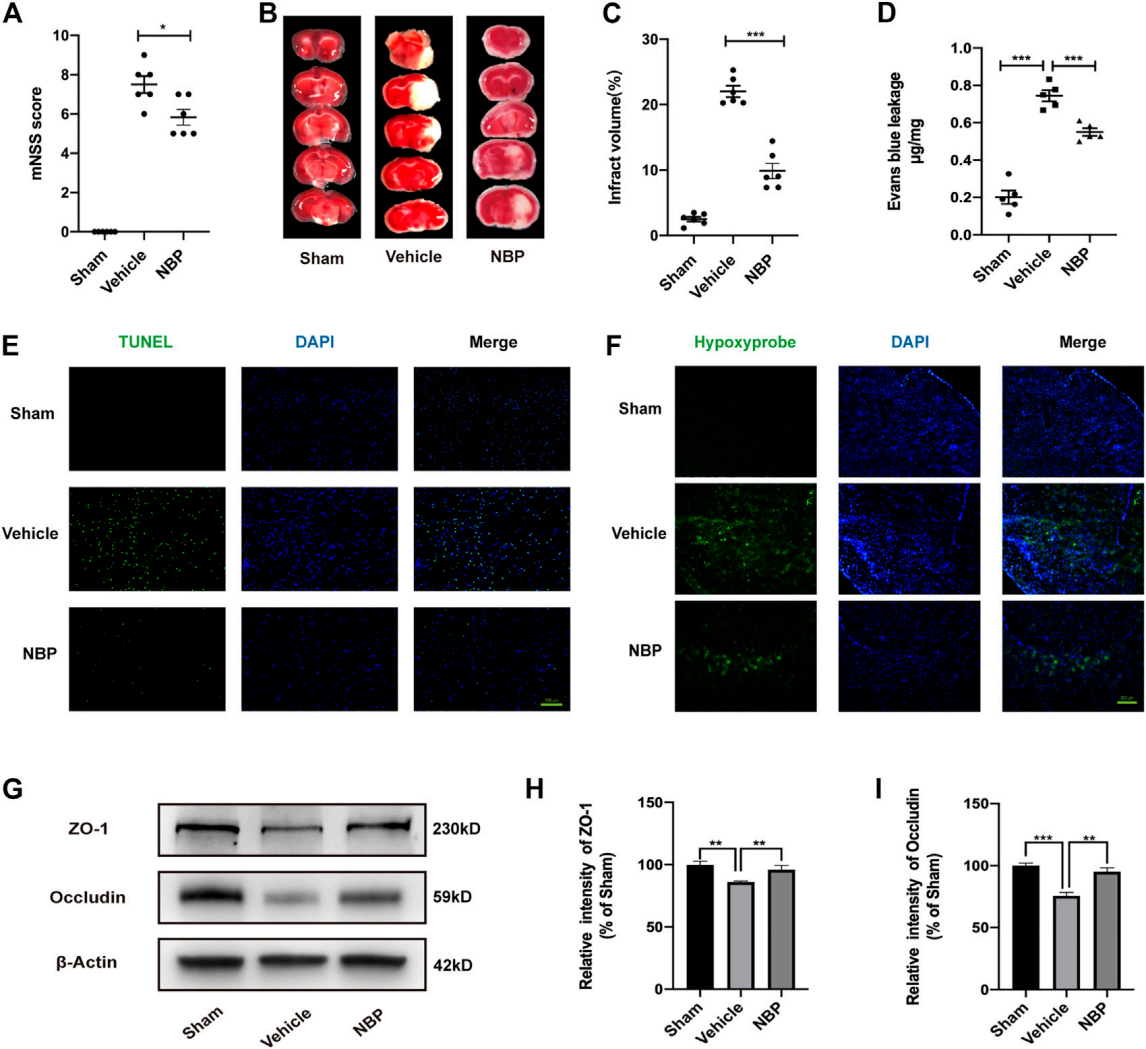
Effects of NBP on damage to neurons and the blood brain barrier after ischemia/reperfusion in mice. **(A)** The mNSS was used to assess neurological deficits. **(B)** TTC staining showed the infarcted area. **(C)** Cerebral infarction volume was calculated and quantified using ImageJ. **(D)** Blood brain barrier permeability was determined based on Evans Blue leakage. **(E)** Inverted fluorescence microscopy showing TUNEL-stained (green fluorescence) apoptotic neural cells. **(F)** Fluorescence microscopy shows the expression of the hypoxyprobe (green fluorescence) in the brain tissue of mice to assess the degree of hypoxia. **(G–I)** Effects of NBP on the protein expression of ZO-1 and occludin in ischemia/reperfusion brain tissue and results of semi-quantitative analysis of protein grey scale. **(A–C)** *n* = 6 mice per group. **(D–F)** *n* = 5 mice per group. **(G–I)** the data are representative of three independent experiments. All data are expressed as mean ± SEM. *p < 0.05, **p < 0.01, ***p < 0.001. Scale bars, **(D)** 100μm, **(F)** 500 μm.

To further perform our quantitative chemical proteomic analysis, we first verified the neuroprotective effect of NBP *in vivo*. Based on previous studies, cerebral ischemia/reperfusion mice were treated with 60 mg/kg corn oil or NBP *via* oral gavage for three consecutive days. Analysis of modified Neurological Severity Scores (mNSS) results indicated that neurological function had improved in the NBP group, relative to that observed in the vehicle group (Figure 1A). We then stained brain sections using 2,3,5-triphenyltetrazolium chloride (TTC) to assess the effect of NBP on the volume of cerebral infarction (Figure 1B). Our results indicated that, in the acute phase, NBP significantly reduced the infarct volume after ischemia/reperfusion (Figure 1C). Leakage of Evans Blue was regarded as an indicator of blood brain barrier permeability (Figure 1D). Terminal deoxynucleotidyl transferase dUTP nick end labelling (TUNEL) staining (Figure 1E) also revealed that neuronal apoptosis was significantly reduced in the NBP group when compared with that in the vehicle group. In order to verify the effect of NBP on brain hypoxia, a hypoxyprobe was used to detect hypoxia in the brains of middle cerebral artery occlusion/reperfusion (MCAO/R) mice. Our findings indicated that NBP significantly reduced brain hypoxia when compared with levels observed in the vehicle group (Figure 1F). Together, these support the notion that NBP exerts neuroprotective effects in mice.

Analysis of the relevant results indicated that NBP treatment significantly reduced blood brain barrier permeability relative to that observed in the vehicle group. Furthermore, although expression of the tight junction proteins (TJs) zonula occludens 1 (ZO-1) and occludin was significantly reduced after cerebral ischemia/reperfusion, these effects were attenuated by NBP treatment (Figures 1G–I). These findings suggest that NBP contributes to maintaining blood brain barrier integrity by increasing the expression of tight junction proteins. Thus, NBP may play an important protective role in vascular endothelial cells during cerebral ischemia/reperfusion. Therefore, vascular endothelial cells were selected as potential target cells for NBP in subsequent experiments.

### Possible protein targets for NBP

**FIGURE 2:**
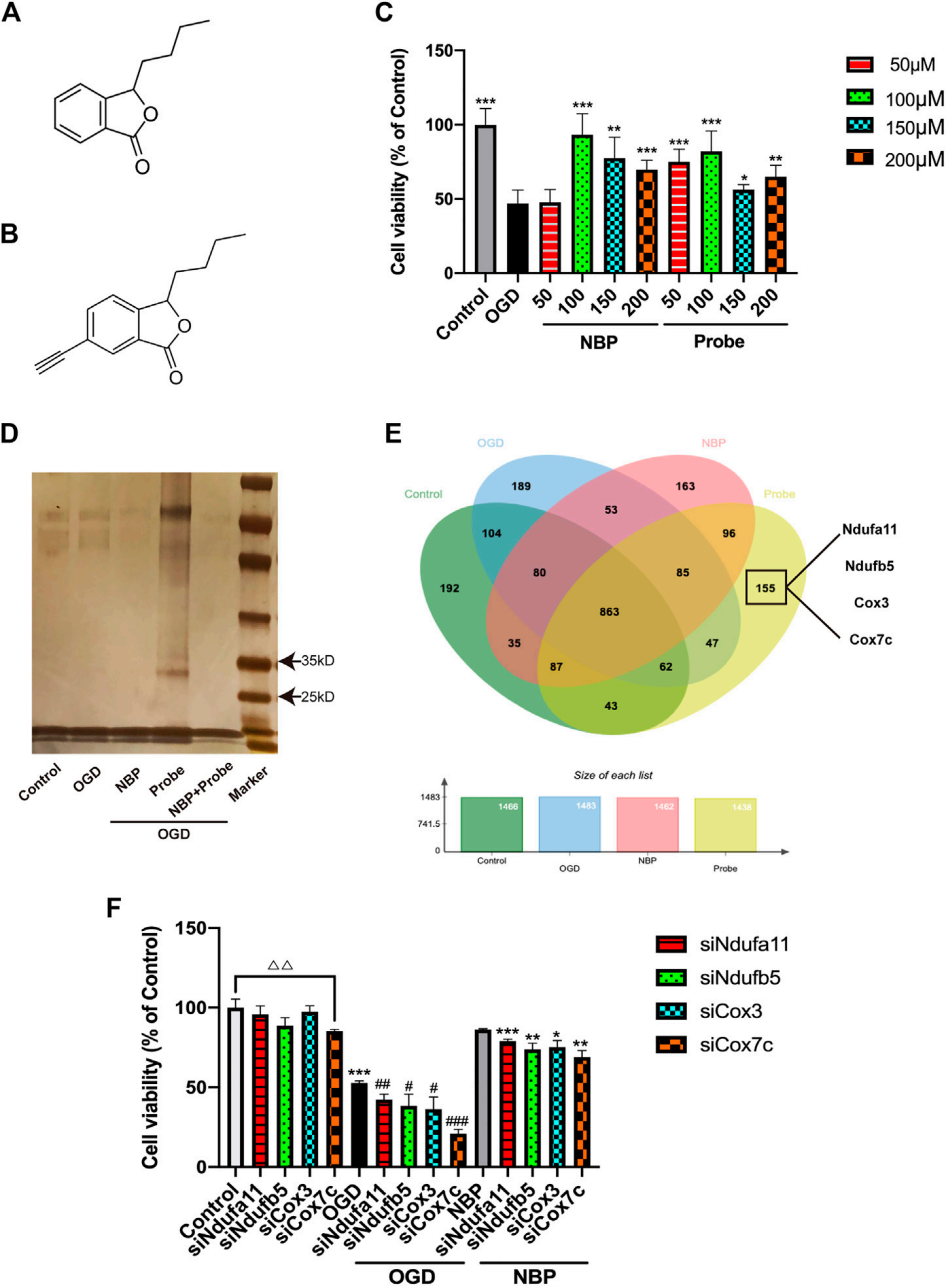
Identification of Cox7c as a possible target of NBP. **(A)** The chemical structure of NBP. **(B)** The chemical structure of the synthesised NBP probe. **(C)** Verification of the influence of NBP and probe on the survival rate of endothelial cells subjected to OGD/R. **(D)** Pulldown experiments were conducted on the OGD/R-induced endothelial cell proteins treated using NBP or probe. After gel electrophoresis, silver staining was conducted to reveal the protein bands that the probe had bound. **(E)** Venn diagram showing the binding proteins after the pulldown experiment (LC-MS/MS). Four proteins related to mitochondrial function were screened out of 155 proteins for further verification. **(F)** Knockdown of the genes corresponding to the four screened proteins by siRNA to verify the influence on cell survival rate after OGD treatment. The data are representative of three independent experiments. All data are expressed as mean ± SEM. **(C)** *p < 0.05, **p < 0.01, ***p < 0.001 compared with OGD group. **(F)** △△*p* < 0.01; *p < 0.05,**p < 0.01, ***p < 0.001 compared with NBP group; #*p* < 0.05, ###*p* < 0.001 compared with OGD group.

We used the bEnd.3 cell line to study the protein targets of NBP. To identify these targets, a probe containing acetylene groups was designed based on the molecular structure of NBP (Figures 2A, B). To verify the role of NBP and the probe in endothelial cells and determine their effective concentration of action, we established an oxygen-glucose deprivation/reperfusion (OGD/R) model of endothelial cells. In these endothelial cells, treatment with NBP or probe both inhibited cell death at a concentration of 100 μM, indicating that the probe may exert similar protective effects. Thus, 100 µM was determined as the working concentration for follow-up experiments (Figure 2C).

The activity-based protein profiling (ABPP) method was used to identify the proteins interacting with NBP in endothelial cells. The bEnd.3 cells were used to construct an OGD/R model, following which they were treated with DMSO, NBP, probe, and NBP + probe, respectively. Extracted proteins were combined with azidobiotin and enriched in streptavidin-sepharose beads. Silver staining of the washed protein revealed that the probe had some specific binding proteins between 25 and 35 kD (Figure 2D). Furthermore, when natural NBP was treated with a synthetic NBP probe, the NBP competed with the probe, supporting the notion that the probe can bind similar target proteins to NBP. We used LC-MS/MS to examine the proteins captured by the probe and ultimately identified 155 proteins as potential targets for specific binding to NBP (Figure 2E). Since the effect of NBP on mitochondrial function has been verified in many studies, we selected four proteins related to mitochondrial function from among the 155 identified proteins for further study, to determine the potential role of these proteins in mediating the effects of NBP on oxidative stress response. We used RNA interference (RNAi) to knock down the four candidate proteins and investigated the cell viability of bEnd.3 cells (Figure 2F). We observed that Cox7c knockdown reduced cell viability and eliminated the beneficial effects of NBP under both normal and OGD conditions. These results suggest that Cox7c is directly related to the inhibitory effect of NBP on vascular endothelial cell apoptosis. Therefore, we hypothesized that NBP inhibits the apoptosis of vascular endothelial cells by directly activating Cox7c.

### NBP upregulates the expression of Cox7c during ischemia/reperfusion

**FIGURE 3:**
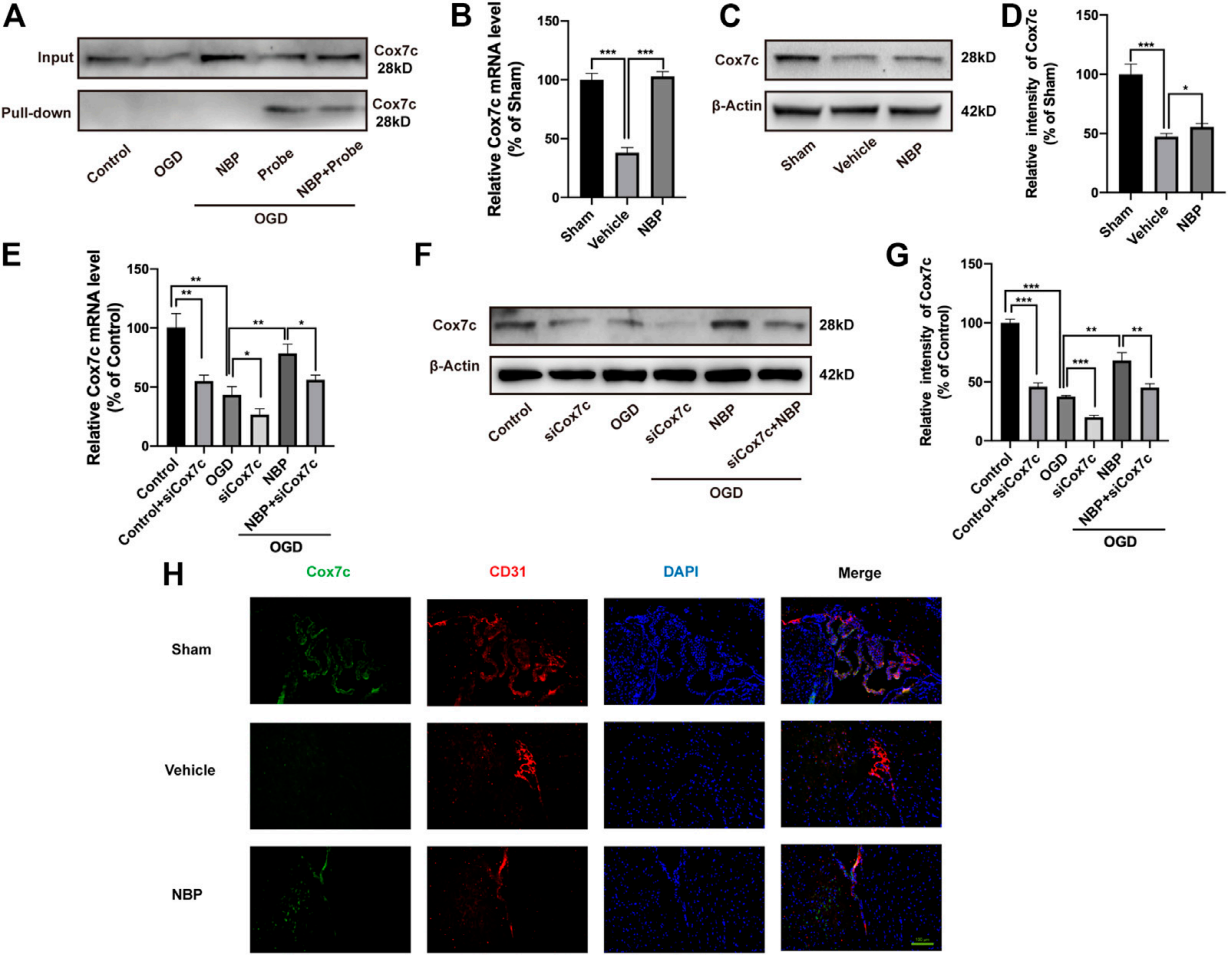
Effects of NBP and siCox7c in ischemia/reperfusion brain tissue and cells. **(A)** Western blotting was used to detect the binding of endothelial cell proteins with and without pulldown to Cox7c after treatment with NBP and probe. **(B)** The effects of NBP on the mRNA expression of Cox7c in MCAO/R mice **(C,D)** The effects of NBP on the protein expression level of Cox7c in MCAO/R mice, and the results of semi-quantitative analysis of protein grey level. **(E)** The effects of NBP on the expression of Cox7c mRNA in OGD/R endothelial cells **(F,G)** The effects of NBP on the expression of Cox7c in OGD/R endothelial cells, and the results of semi-quantitative analysis of protein grey level. **(H)** Fluorescence microscopy shows the expression of Cox7c (green fluorescence) and CD31 (red fluorescence) in the brain tissue of MCAO/R mice. The data are representative of three independent experiments. All data are expressed as mean ± SEM. *p < 0.05, **p < 0.01, ***p < 0.001. Scale bars, **(H)** 100 μm.

We verified the mutual binding between NBP and Cox7c, following which the click-reaction-assisted activity-based protein profiling(ABPP) method was used for the pulldown of the proteins. Western blotting experiments revealed that, in bEnd.3 cells, the probe could combine with Cox7c and that such a combination was reduced after co-treatment with the probe and NBP (Figure 3A). This result indicates that NBP can competitively inhibit the binding of the probe to Cox7c, supporting the notion that mutual binding can occur between Cox7c and NBP. Next, we investigated the mRNA and protein expression of Cox7c in brain tissues from MCAO/R mice. These experiments revealed that NBP promoted the expression of Cox7c when compared with that observed in the vehicle group (Figures 3B–D). Further analysis revealed that levels of Cox7c mRNA and protein expression were significantly decreased in bEnd.3 cells subjected to OGD. However, NBP increased the expression of Cox7c, while siCox7c offset the upregulating effect of NBP (Figures 3E–G). We also investigated the expression of Cox7c and the endothelial marker CD31 in mouse brain tissue *via* immunofluorescence staining, observing that Cox7c was widely expressed in vascular endothelial cells. In contrast, the expression of Cox7c was significantly decreased in vascular endothelial cells subjected to cerebral ischemia, and the expression of Cox7c was upregulated by NBP treatment (Figure 3H). We also performed immunofluorescence staining for Cox7c in bEnd.3 cells and labelled the mitochondria using Mitotracker (Supplementary Figure S1). We observed that the localization of Cox7c was consistent with that of mitochondria, and that the trend of Cox7c expression in mitochondria was consistent with the results of quantitative polymerase chain reaction (PCR) and Western blotting experiments. This result indicates that Cox7c may be associated with mitochondrial function.

### NBP inhibits mitochondrial apoptosis *via* upregulation of Cox7c

TUNEL and Mitotracker staining revealed that OGD/R significantly increased the number of apoptotic bEnd.3 cells, consistent with the results of mitochondrial localization experiments. Further increases in the number of apoptotic cells were observed following OGD and siCox7c treatment. NBP treatment reduced the number of apoptotic cells, while siCox7c inhibited the anti-apoptotic effect of NBP (Supplementary Figure S2).

**FIGURE 4:**
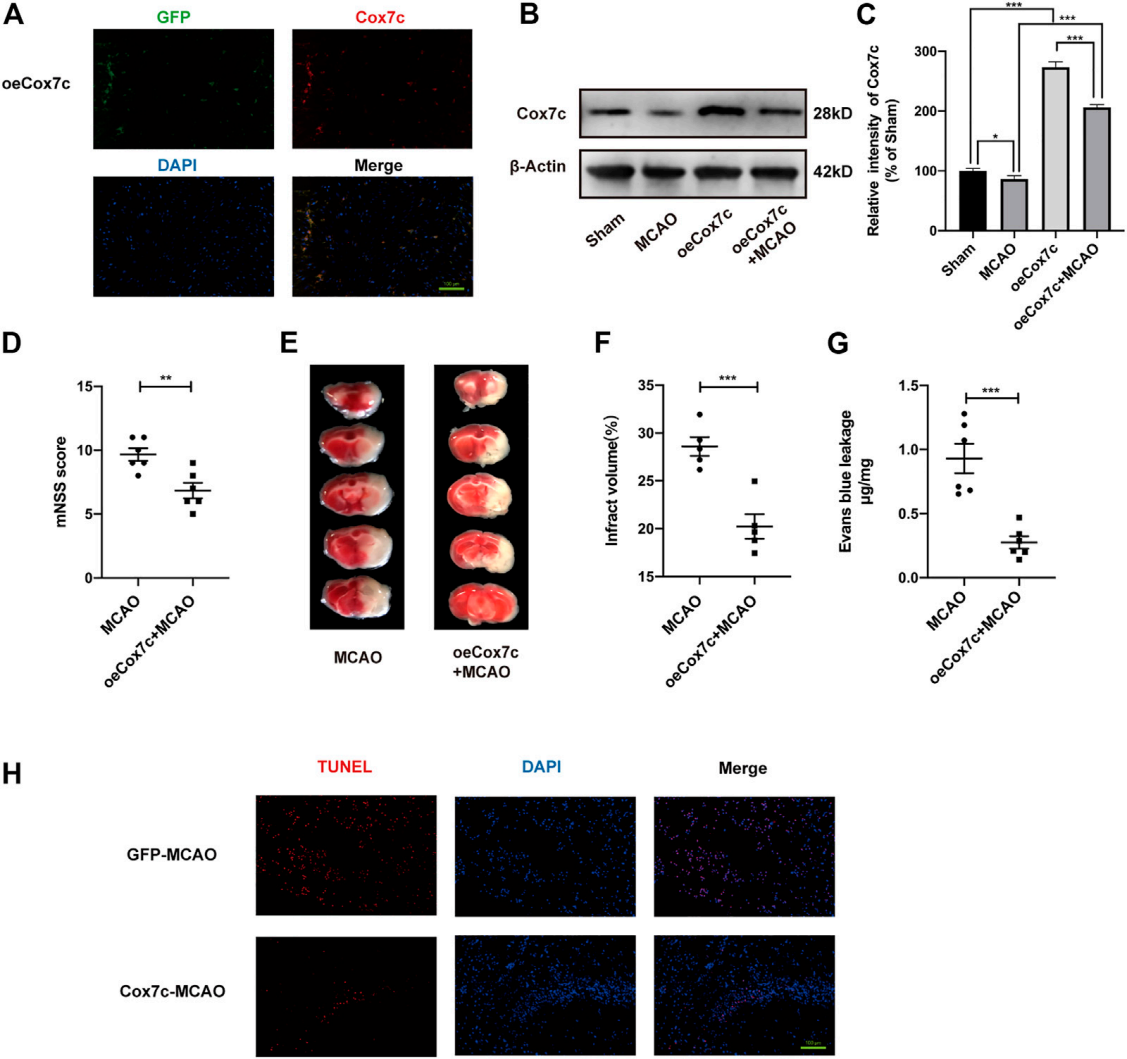
Effects of oeCox7c on ischemia/reperfusion models *in vivo*. **(A)** Fluorescence microscopy showing Cox7c (red fluorescence) expression in oeCox7c mouse brain and co-localisation with GFP (green fluorescence) **(B,C)** Effects of oeCox7c on Cox7c expression in MCAO/R mouse brain, and results of semi-quantitative analysis of protein grey scale. **(D)** The mNSS was used to assess neurological deficits of control and oeCox7c MCAO/R mice. **(E)** TTC staining shows the infarcted area of control and oeCox7c MCAO/R mice. **(F)** Cerebral infarction volume was calculated and quantified using ImageJ. **(G)** Blood brain barrier permeability was determined based on Evans Blue leakage of control and oeCox7c MCAO/R mice. **(H)** Inverted fluorescence microscopy showing TUNEL-stained (green fluorescence) apoptotic neural cells. The data are representative of three independent experiments. All data are expressed as mean ± SEM. *p < 0.05, **p < 0.01, ***p < 0.001. Scale bars, **(A,H)**:100 μm.

We also further validated the mechanism of Cox7c against ischemia/reperfusion damage by overexpressed Cox7c in MCAO/R mice and OGD/R-induced bEnd.3 cells. We overexpressed Cox7c (oeCox7c) *in vivo* and *in vitro* through lentivirus and AAV vectors and detected the effects of overexpression *via* fluorescence staining and Western blotting. Our results showed that oeCox7c increased expression of Cox7c on vascular endothelial cells *in vivo* and *in vitro* (Figures 4A–C; Supplementary Figures S3A, B). The mNSS scores suggested that oeCox7c improved the neurological function of MCAO/R mice (Figure 4D). The results of TTC staining indicated that oeCox7c reduced the volume of cerebral infarction after ischemia/reperfusion (Figures 4E, F). Leakage of Evans Blue represented oeCox7c treatment significantly reduced blood brain barrier permeability relative to the MCAO/R group (Figure 4G). TUNEL staining revealed that oeCox7c reduced the apoptosis of neuronal cells in MCAO/R mice (Figure 4H).

In addition, we detected the expression of TJs *in vivo* and *in vitro*, the results show that OGD and siCox7c inhibited the expression of ZO-1 and occludin, while NBP increased such expression. However, siCox7c attenuated the effects of NBP (Supplementary Figures S4A–C). Detection of TJs also showed that increased expression of ZO-1 and occludin in oeCox7c models, compared to the control group, and oeCox7c also increase these expressions in ischemia/reperfusion models *in vivo* and *in vitro* (Supplementary Figures S4D–I).

### NBP protects mitochondrial function *via* upregulation of Cox7c

**FIGURE 5:**
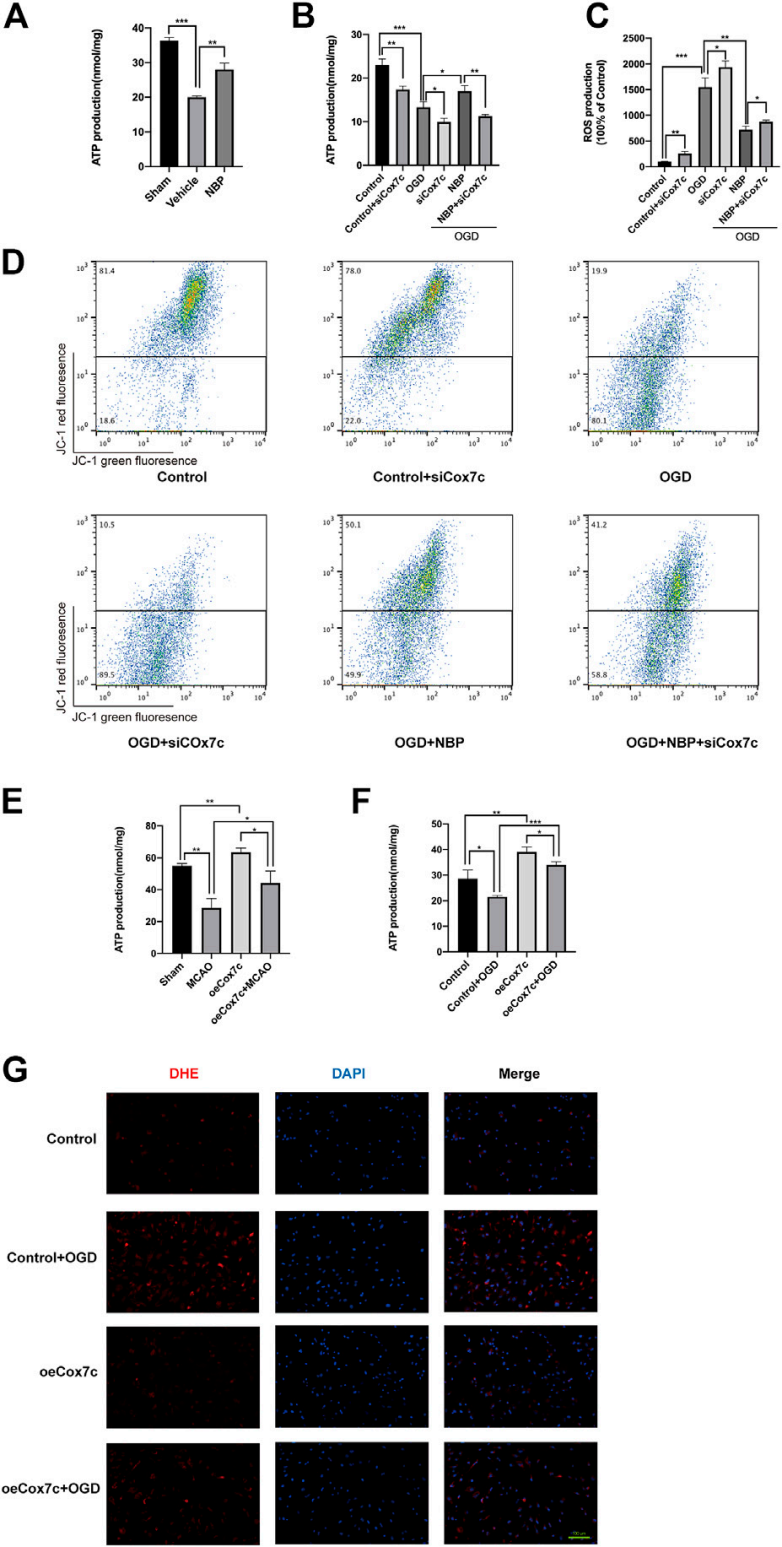
Effects of NBP and Cox7c on mitochondrial function *in vivo* and *in vitro*. **(A)** The effects of NBP on ATP production in MCAO/R mice brains. **(B)** The effects of NBP and siCox7c on ATP production in OGD/R-induced bEnd.3 cells. **(C)** The effects of NBP and siCox7c on ROS production in OGD-induced bEnd.3 cells were detected *via* DHE staining and detected by flow cytometry. **(D)** The effects of NBP and siCox7c on the mitochondrial membrane potential of OGD/R-induced bEnd.3 cells were detected *via* flow cytometry. **(E)** The effects of oeCox7c on ATP production in MCAO/R mice brains. **(F)** The effects of oeCox7c on ATP production in OGD/R-induced bEnd.3 cells. **(G)** The effects of oeCox7c on ROS (red fluorescence) production in OGD-induced bEnd.3 cells were detected *via* DHE staining. The data are representative of three independent experiments. All data are expressed as mean ± SEM. *p < 0.05, **p < 0.01, ***p < 0.001. Scale bars, **(G)**100 μm.

We used an ATP assay kit to detect ATP production in brain tissue and endothelial cells, respectively. *In vivo*, ATP content was significantly lower in the vehicle group than in the sham group, while NBP treatment increased ATP production (Figure 5A). Similar results were obtained in endothelial cells: ATP production was reduced in the OGD group; NBP promoted ATP production; and the application of siCox7c reduced the production of ATP in the control, OGD, and NBP treatment groups (Figure 5B). Dihydroethidine (DHE) staining and flow cytometry were used to examine ROS production, which was higher in endothelial cells subjected to OGD than in controls. SiCox7c further increased ROS production, while NBP significantly inhibited ROS production in endothelial cells (Figure 5C). Flow cytometry was also used to detect the mitochondrial membrane potential of endothelial cells stained with JC-1. We found that OGD significantly decreased mitochondrial membrane potential, while NBP inhibited this change. Cox7c knockdown reduced the mitochondrial membrane potential of endothelial cells in the control, OGD, and NBP treatment groups (Figure 5D).

We have also detected the effect of oeCox7c on mitochondrial function. *In vivo*, the production of ATP in the oeCox7c group was significantly higher than that of the sham group, and oeCox7c elevated the production of ATP in MCAO/R mice (Figure 5E). *In vitro*, oeCox7c increased the production of ATP in control or OGD/R-induced bEnd3 cells (Figure 5F). The mitochondria of bEnd.3 cells were marked by Mitotracker, which revealed that oeCox7c significantly restrained reduction in the number of mitochondria in OGD/R-induced bEnd3 cells (Fig.S5). The DHE staining showed that oeCox7c reduced the production of ROS in OGD/R-induced bEnd3 cells (Figure 5G).

### NBP protects mitochondrial respiration *via* upregulation of Cox7c

**FIGURE 6:**
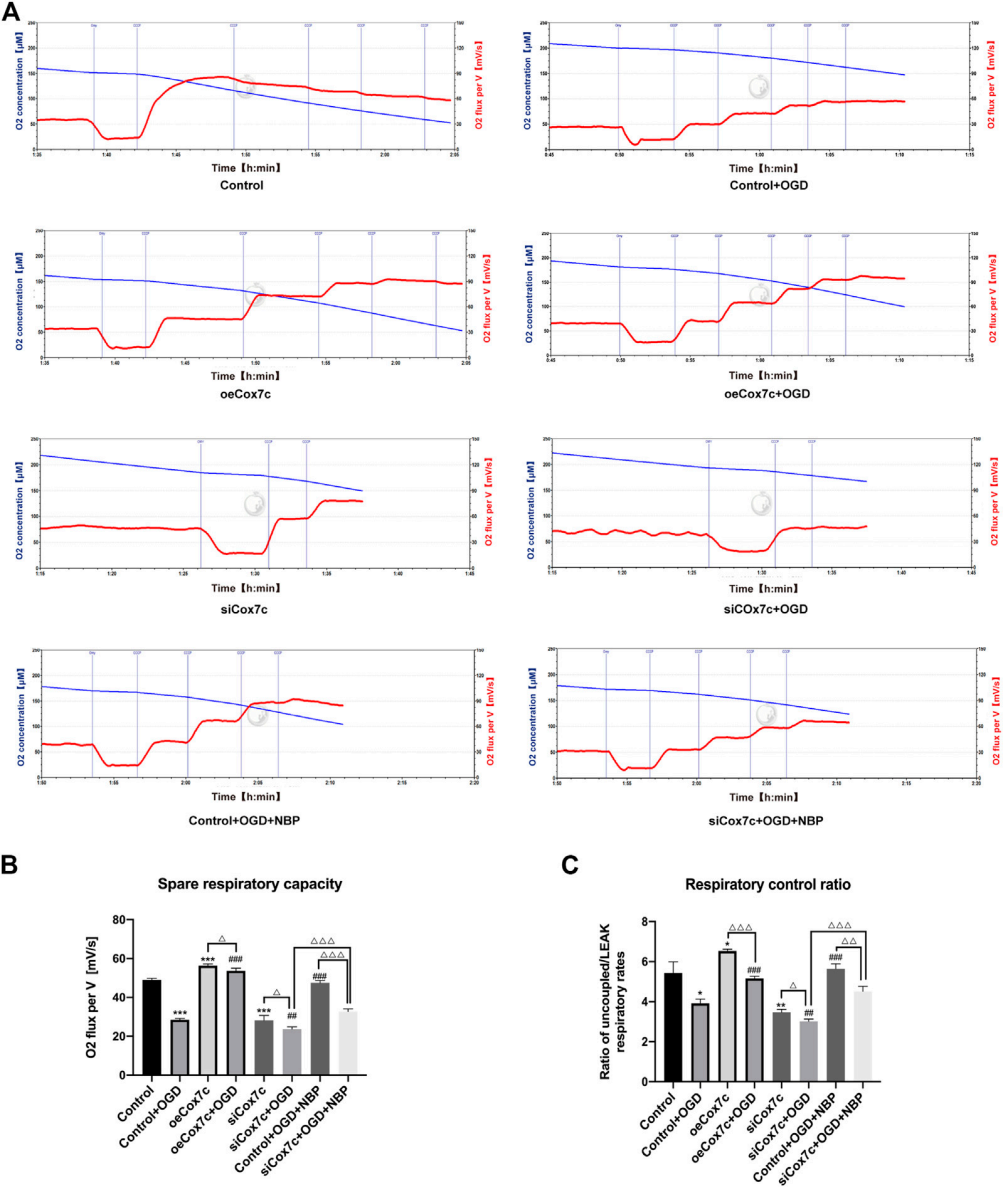
Effects of NBP, siCox7c and oeCox7c(overexpression of Cox7c) on mitochondrial respiration in OGD/R-induced bEnd.3 cells. **(A)** The O_2_ flux of bEnd.3 cells were measured by Oroboros O2K, with subsequent additions of oligomycin (Omy) and CCCP. The blue line indicates the level of O_2_ concentration (μM, left Y-axis) and the red line indicates the oxygen consumption rate (O_2_ flux per V[mV/s], right Y-axis). **(B)** The spare respiratory capacity was calculated and quantified using Oroboros O2k. **(C)** The respiratory control ratio was calculated and quantified using Oroboros O2k. The data are representative of three independent experiments. All data are expressed as mean ± SEM. *p < 0.05, **p < 0.01, ***p < 0.001 compared with the Control group; #*p* < 0.05, ##*p* < 0.01 compared with the Control + OGD group; the remaining differences are marked above the connecting line between the two groups, ΔP < 0.05, ΔΔP <0.01, ΔΔΔP <0.001.

The Oroboros O2K was used to measure oxygen consumption rates of bEnd.3 cells in each group. The routine respiration was measured when O2K stabilized, LEAK respiration was detected after injection of oligomycin (Omy), and the maximum respiration was determined by sequential injection of CCCP to reached a plateau (Figure 6A). We defined the difference of oxygen consumption rates between the maximum respiration and routine respiration as the spare respiratory capacity, which represents the spare capacity of ATP generation. Besides, the respiration rates ratio of maximum respiration to LEAK respiration was defined as the respiratory control ratio, which represents the respiration capacity of mitochondria. Our results displayed that the spare respiratory capacity of the OGD group and siCox7c group significantly decreased, and the oeCox7c group increased compared to the control group, while oeCox7c and NBP reduced the degree to which spare respiratory capacity OGD suppressed (Figure 6B). In addition, compared to the control group, respiratory control ratio in OGD-induced bEnd.3 cells was significantly reduced, and siCox7c had a similar effect, while oeCox7c increased the respiratory control ratio (Figure 6C).

### NBP promotes HIF-1α/VEGF pathway *via* upregulation of Cox7c

**FIGURE 7:**
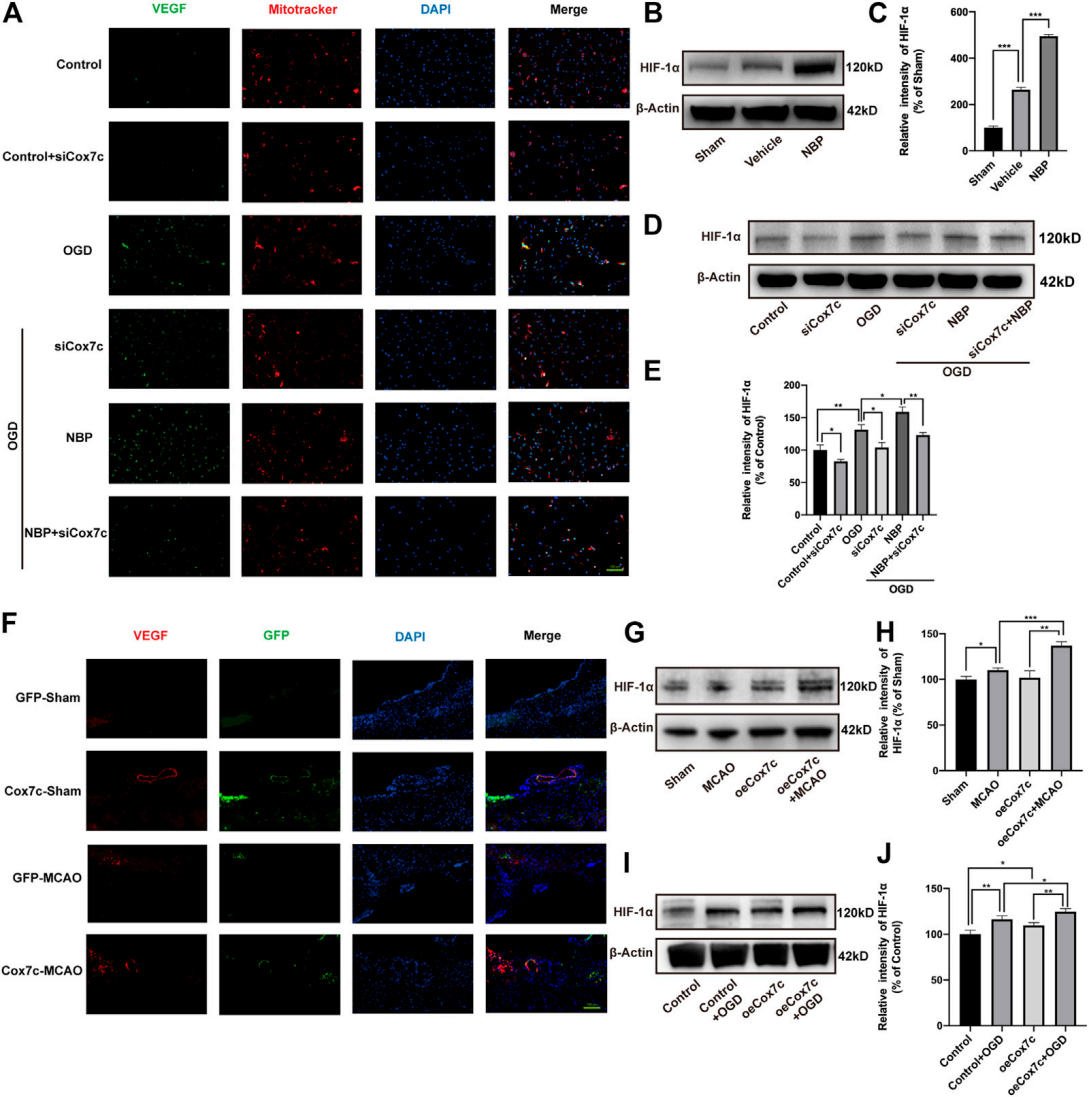
Effects of NBP, siCox7c and oeCox7c on VEGF/HIF-1α pathway in vascular endothelial cells after ischemia/reperfusion injury. **(A)** Fluorescence inverted microscopy shows the expression of VEGF (green fluorescence) and Mitotracker (red fluorescence) in OGD/R-induced bEnd.3 cells. (**B,C**) Effect of NBP on HIF-1α expression in MCAO/R mouse brain, and results of semi-quantitative analysis of protein grey scale **(D,E)** Effects of NBP and siCox7c on HIF-1α expression in OGD/R-induced bEnd.3 cells, and results of semi-quantitative analysis of protein grey scale. **(F)** Fluorescence inverted microscopy showing the expression of VEGF (red fluorescence) and GFP (green fluorescence) in the brain tissue of mice. **(G,H)** Effect of oeCox7c on HIF-1α expression in MCAO/R mouse brain, and results of semi-quantitative analysis of protein grey scale **(I,J)** Effects of oeCox7c on HIF-1α expression in OGD/R-induced bEnd.3 cells, and results of semi-quantitative analysis of protein grey scale. The data are representative of three independent experiments. All data are expressed as mean ± SEM. *p < 0.05, **p < 0.01, ***p < 0.001. Scale bars, **(A,F)**:100 μm.

Immunofluorescence and Mitotracker experiments indicated that OGD increased the expression of VEGF, while siRNA reduced the expression of vascular endothelial growth factor (VEGF). When compared with that in the OGD group, NBP significantly increased the expression of VEGF, while siCox7c inhibited the upregulating effect of NBP on VEGF (Figure 7A). We detected hypoxia-inducible factor 1α (HIF-1α) expression *via* Western blotting both *in vitro* and *in vivo*. *In vivo*, NBP significantly increased HIF-1α expression when compared with vehicle treatment (Figures 7B, C). *In vitro*, HIF-1α expression was significantly increased in OGD-induced endothelial cells. SiCox7c downregulated HIF-1α expression, while NBP treatment increased HIF-1α expression (Figures 7D, E). We also examined the mRNA expression of VEGFA and VEGFB in mice and bEnd.3 cells, observed that levels of both were upregulated following cerebral ischemia/reperfusion. NBP treatment further increased the expression of VEGFA and VEGFB (Supplementary Figures S6A, B). Similar results were obtained in endothelial cells, and siCox7c inhibited the ability of NBP to increase the mRNA expression of VEGFA and VEGFB (Supplementary Figures S6C, D).

For oeCox7c, we found that oeCox7c significantly improved the expression of VEGF in mouse brain using immunofluorescent staining, especially in vascular endothelial cells (Figure 7F). We also measured the expression of HIF-1α, similar to previous results, the expression of HIF-1α increased in ischemia/reperfusion compared to the control group *in vivo* and *in vitro*, while oeCox7c further increased the expression of HIF-1α (Figures 7G–J).

## Discussion

In the present study, we investigated NBP and protein interactions in *in vivo* and *in vitro* models of acute ischemic stroke using a quantitative chemical proteomics approach. Our results indicated that NBP improves neurological symptoms, reduces cerebral infarction area, and inhibits neuronal cell apoptosis in ischemia/reperfusion mice. In addition, NBP reduced EB leakage, suggesting a protective effect against blood brain barrier damage. Endothelial cells help form the blood brain barrier through TJs, limiting the entry of many compounds and harmful substances into the brain. Ischemia/reperfusion injury causes the destruction of TJ, leading to excessive blood brain barrier permeability and angio-cerebral oedema(Jiang et al., 2018). ZO-1 can bind to the distal C- terminus of occludin to form a transmembrane tight junction protein, and thus is closely related to the integrity of blood brain barrier(Erickson et al., 2007). Our findings indicated that, although ischemia/reperfusion injury decreased the expression of ZO-1 and occludin, application of NBP increased such expression. This evidence supports the notion that NBP contributes to the maintenance of TJs and protects the integrity of cerebrovascular endothelial cells comprising the blood brain barrier.

Despite the growing number of studies on the role of NBP in ischemic stroke, no specific conclusions have been drawn regarding the combined targets of NBP. In the present study, we sought to identify specific target proteins of NBP by adding an alkynyl group to the NBP molecule and synthesizing an NBP probe. Based on the results of this experiment, we used the mouse vascular endothelial cell line bEnd.3 to construct an *in vitro* model of ischemia/reperfusion. To verify whether the acetylenyl group changes the effect of NBP on cells, we examined the effects of different NBP and probe concentrations on cell viability in OGD/R-induced endothelial cells. Our findings demonstrated that both the probe and NBP exerted similar effects on cell viability. Subsequent experiments utilized a concentration of 100 μM. After pulldown, silver staining results revealed that there was direct protein binding to the probe between 25 and 35 kD, which may have been inhibited by competitive binding of NBP. Protein spectrogram analysis in the control, OGD, NBP, and probe groups allowed us to identify 155 different proteins. Given that previous research has indicated that NBP may exert its neuroprotective effects by protecting mitochondrial function, maintaining mitochondrial membrane potential, and increasing COX levels (Lv et al., 2018), we selected the following four mitochondrial function-related proteins for further analysis: Ndufa11, Ndufb5, Cox3, and Cox7c. RNA interference experiments revealed that only Cox7c expression decreased in the control, OGD, and NBP groups, suggesting that Cox7c is among the key binding targets of NBP.

Cox7c is a main component of COX, the terminal enzyme of the electron transport chain responsible for mitochondrial respiration. In this chain, cytC provides electrons that catalyse the reduction of oxygen into water (Cooper et al., 1991; Brunori and Wilson 1995; Hofmann et al., 1998). COX is localized on the mitochondrial membrane, where some subunits encode in mitochondrial DNA and others in the nucleus. Cox7c encodes in the nucleus, and the synthesized protein is translocated to the mitochondria and binds to COX complexes on the mitochondrial membrane (Schatz 1974; Cumsky et al., 1983). Studies have demonstrated that the downregulation of Cox7c is characteristic of chronic kidney disease, supporting the notion that Cox7c is involved in the regulation of mitochondrial function and oxidative stress (Zaza et al., 2013). A recent study found that the expression of Cox7c was correlated with venous thromboembolism in patients with colon cancer (Wu et al., 2020). Other studies have reported that upregulation of Cox7c is associated with the occurrence of some tumors, such as skin cancer, breast cancer, and bladder cancer (Dang et al., 2006; Marsit et al., 2011; Wang et al., 2017). However, rare research reported the role of Cox7c in cerebral ischemia/reperfusion currently, therefore we conducted a series of studies focusing on Cox7c.

Our pulldown experiment verified that the probe could directly bind to Cox7c and could be competitively inhibited by NBP, suggesting that NBP directly binds to Cox7c. Further analysis indicated that ischemia/reperfusion injury resulted in decreased expression of Cox7c, while NBP increased the expression of Cox7c. We found that Cox7c was widely expressed in vascular endothelial cells and that changes in mRNA expression levels were consistent with changes in protein levels. Co-localisation of Cox7c with mitochondria in endothelial cells indicated that Cox7c was mainly expressed in mitochondria. This is consistent with our expectation that Cox7c is likely to affect vascular endothelial cells by affecting mitochondrial function.

Mitochondria are believed to be the main source of ischemia/reperfusion-induced ROS production in various organs, especially in the heart and brain, which exhibit high metabolic activity (Sanderson et al., 2013; Kalogeris et al., 2014; Zorov et al., 2014). During cerebral ischemia/reperfusion, especially in the reperfusion stage, the production of ROS increases significantly. Due to the lack of oxygen and glucose in brain tissue, ATP production is reduced and calcium concentration in neurons is increased, which leads to mitochondrial depolarisation and the production of a large amount of ROS (Dröse and Brandt 2012; Kalogeris et al., 2014; Shenoda 2015). One molecule of glucose produced 31 ATP molecules by mitochondrial oxidative phosphorylation, while two ATP molecules were generated by anaerobic glycolysis under hypoxia conditions (Zepeda et al., 2013). Consistent with the findings of previous studies, our results indicate that ischemia/reperfusion plays a key role in promoting apoptosis by reducing mitochondrial membrane potential, reducing ATP synthesis, and inducing ROS production (Heo et al., 2005; Sanderson et al., 2013). By binding to Cox7c, NBP alleviates tissue hypoxia and maintains the stability of mitochondrial membrane potential, thereby reduced anaerobic glycolysis and increased ATP production during oxidative phosphorylation, while knockdown of Cox7c inhibited this effect. As previously mentioned, Cox7c is a component of COX, which plays a critical role in maintaining mitochondrial membrane potential and to promote ATP generation during oxidative phosphorylation. We used O2K instruments to determine the effect of NBP, siCox7c, oeCox7c on the mitochondrial respiration capacity of vascular endothelial cells, and this result also proved our conclusions. We found that the oxygen consumption rate of mitochondria was inhibited after OGD/R, which was increased by NBP. Our experiments of up-regulating and down-regulating Cox7c respectively demonstrated that Cox7c can significantly increase the rate of oxygen consumption after OGD/R-induced damage. We performed a statistical analysis of spare respiratory capacity and respiratory control ratio, and our results shown that NBP significantly increased spare respiratory capacity and respiratory control ratio by upregulating Cox7c, suggesting an increase in oxygen store in the mitochondria for the production of ATP, thereby promoting ATP synthesis and improving mitochondrial respiration capacity. Our results illustrated the protective effect of NBP on the comprehensive function of mitochondria *via* Cox7c. Therefore, our findings suggest that NBP increases ATP production and maintains mitochondrial membrane potential by increasing the expression of Cox7c.

Consistent with the results of *in vivo* experiments, we also observed that ZO-1 and occludin expression in endothelial cells decreased significantly after OGD induction, while NBP treatment increased such expression. The overexpression experiments *in vivo* and *in vitro* demonstrated that Cox7c increased the expression of ZO-1 and occludin in endothelial cells, demonstrated the effect of Cox7c in inhibiting damage in TJs. Knockdown of Cox7c also significantly inhibited the upregulation of ZO-1 and occludin by NBP, indicating that NBP may inhibit the injury of TJs *via* upregulation of Cox7c expression in endothelial cells, consequently protecting the integrity of blood brain barrier. Studies have shown that ZO-1 and occludin are part of the activated signal nodes of VEGF, and specifically regulate vascular endothelial cell proliferation during angiogenesis (Harhaj et al., 2006; Chidiac et al., 2016). Previous studies have demonstrated that VEGF rapidly increased the phosphorylation and extraction of ZO-1 and occludin, which may contribute to regulated vascular permeability (Chidiac et al., 2016). The increased VEGF levels due to hypoxia were positively correlated with changes in TJ redistribution of ZO-1 and occludin, as well as changes in the actin cytoskeleton both *in vivo* and *in vitro *(Yang et al., 2018). However, the exact mechanism by which HIF-1 regulates TJ proteins remains to be fully elucidated. We will further confirm the mechanism of Cox7c affecting TJ proteins by regulating HIF-1α/VEGF in subsequent studies. The HIF-1α/VEGF signaling pathway reduces apoptosis caused by cerebral ischemia/reperfusion by regulating cell metabolism, angiogenesis, and cell death to cellular adaptation to hypoxia (Dong et al., 2014). Under constant oxygen conditions, HIF-1α is rapidly ubiquitinated and degraded. However, under hypoxic conditions, HIF-1α is stably expressed and upregulated (Jośko and Mazurek 2004; Déry et al., 2005). Previous studies have demonstrated that inhibitor of COX leads to inhibit mitochondrial respiration, and inhibits the accumulation and stabilization of HIF-1α in hypoxia (Hagen et al., 2003; Taylor and Moncada 2010). Therefore, when Cox7c is overexpressed, it resisted the destructive effect of hypoxia on the mitochondrial respiratory chain, and also promoted the stability and expression of HIF-1α. Angiogenesis is an important intrinsic self-protective mechanism after injury. VEGF is a specific mitogen of endothelial cells and an important regulator of angiogenesis (Ferrara and Davis-Smyth 1997). HIF-1α is a major regulator of ischemic angiogenesis, mainly determining oxidative regulatory activity, and plays an important role in various pathophysiological processes (Wang et al., 1995). Our results proved that NBP can further increase the expression of HIF-1α after ischemia/reperfusion, while knockdown Cox7c can inhibit the expression of HIF-1α. This change trend is consistent with VEGF, suggesting that NBP may induce the expression of HIF-1α by upregulating Cox7c, thereby activating VEGF and inducing angiogenesis. VEGF is involved in vasculogenesis and angiogenesis, which helps brain tissue restore oxygen supply, promotes neurotrophic signaling, and regulates the expression of pro-apoptotic and anti-apoptotic factors to produce neuroprotective effects (Uthman et al., 2018; Abdel-Latif et al., 2020). Our study found that NBP can increase VEGF expression in the brain and endothelial cells after ischemia/reperfusion, suggesting that NBP helps to promote angiogenesis. The expression of VEGF increased in oeCox7c groups, while knockdown of Cox7c counteracted the increased effect of NBP on VEGF expression.

Our study still has certain limitations. We screened out four mitochondrial-related proteins that bound to NBP, in which Cox7c was selected as the object of this study, and its neuroprotective effect was proved. Ndufa11, Ndufb5, Cox3 also exhibited varying degrees of inhibition of apoptosis in vascular endothelial cells, therefore NBP may also have other directly bound mitochondrial targets, which need to be further studied. Our study demonstrated the inhibition of the mitochondrial apoptosis pathway and the upregulation of the HIF-1α/VEGF pathway by Cox7c, resulting in a neuroprotective effect during cerebral ischemia/reperfusion. However, it is unclear whether Cox7c affects other pathways, such as the mitochondrial autophagy pathway, which is the direction of our next further research. Through our research, we found that Cox7c is able to reduce the production of ROS and inhibit mitochondrial oxidative stress. Therefore, we speculated suspect that oeCox7c may reduce endothelial apoptosis after cerebral ischemia/reperfusion by inhibiting the mitochondrial apoptosis pathway, but this specific mechanism needs to be further studied and verified, which is also the content of our subsequent research.

**FIGURE 8:**
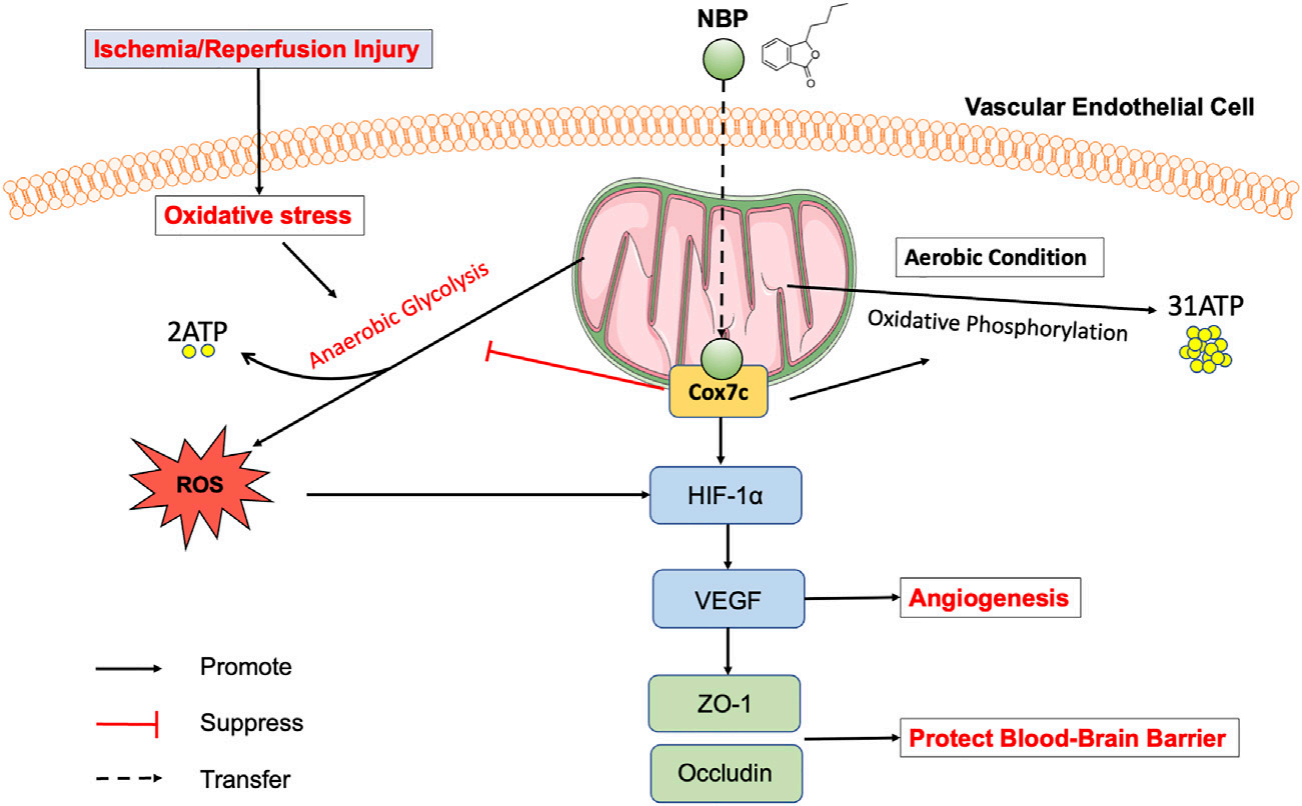
Model of the mechanism by which NBP inhibits mitochondrial dysfunction in ischemia/reperfusion injured vascular endothelial cells. Ischemia/reperfusion induces mitochondrial dysfunction in vascular endothelial cells, while NBP can directly target Cox7c on mitochondria, inhibits anaerobic glycolysis, increases the production of ATP by upregulated the expression of Cox7c, promotes angiogenesis by upregulated the HIF-1α/VEGF pathway, increases the expression of TJs (ZO-1, occludin) to protect blood brain barrier. In addition, NBP inhibits the release of ROS, and then protects the mitochondrial membrane potential, inhibits oxidative stress response, suppresses the apoptosis pathway.

In conclusion, our study proposed for the first time the possible beneficial effects of Cox7c as a key target of NBP in the processes of cerebral ischemia and injury repair. NBP was observed to attenuate impairments in neural function caused by ischemia/reperfusion, reduce the extent of cerebral infarction, and protect the integrity of the blood brain barrier (Figure 8). *In vitro* and *in vivo* experiments further revealed that NBP increases the expression of Cox7c *via* direct binding. This results in increased production of ATP, maintenance of the mitochondrial membrane potential, inhibition of the mitochondrial apoptosis pathway, promotion of endothelial cell angiogenesis, and inhibition of tissue injury caused by hypoxia. Our results also suggest that, by promoting the expression of Cox7c, NBP reduces the production of ROS, and inhibits oxidative stress reactions. Together, our data indicate that Cox7c plays a critical role in the process of ischemia/reperfusion injury and that NBP exerts its neuroprotective effects by promoting the expression of Cox7c in cerebral vascular endothelial cells. At present, studies regarding the role of Cox7c in cerebral ischemia/reperfusion are still rare. Our study provides new insight into the mechanisms underlying ischemia/reperfusion injury and may aid in the design of future studies investigating the application of NBP in the treatment of ischemic stroke.

## Data Availability

The original contributions presented in the study are included in the article/Supplementary Material further inquiries can be directed to the corresponding author.
