# Supplementary figures and images for: Effect of Dl-3-n-butylphthalide on mitochondrial Cox7c in models of cerebral ischemia/reperfusion injury
Source: Front Pharmacol. 2023 Feb 22;14:1084564. doi: 10.3389/fphar.2023.1084564 (PMC9992206; doi:10.3389/fphar.2023.1084564)

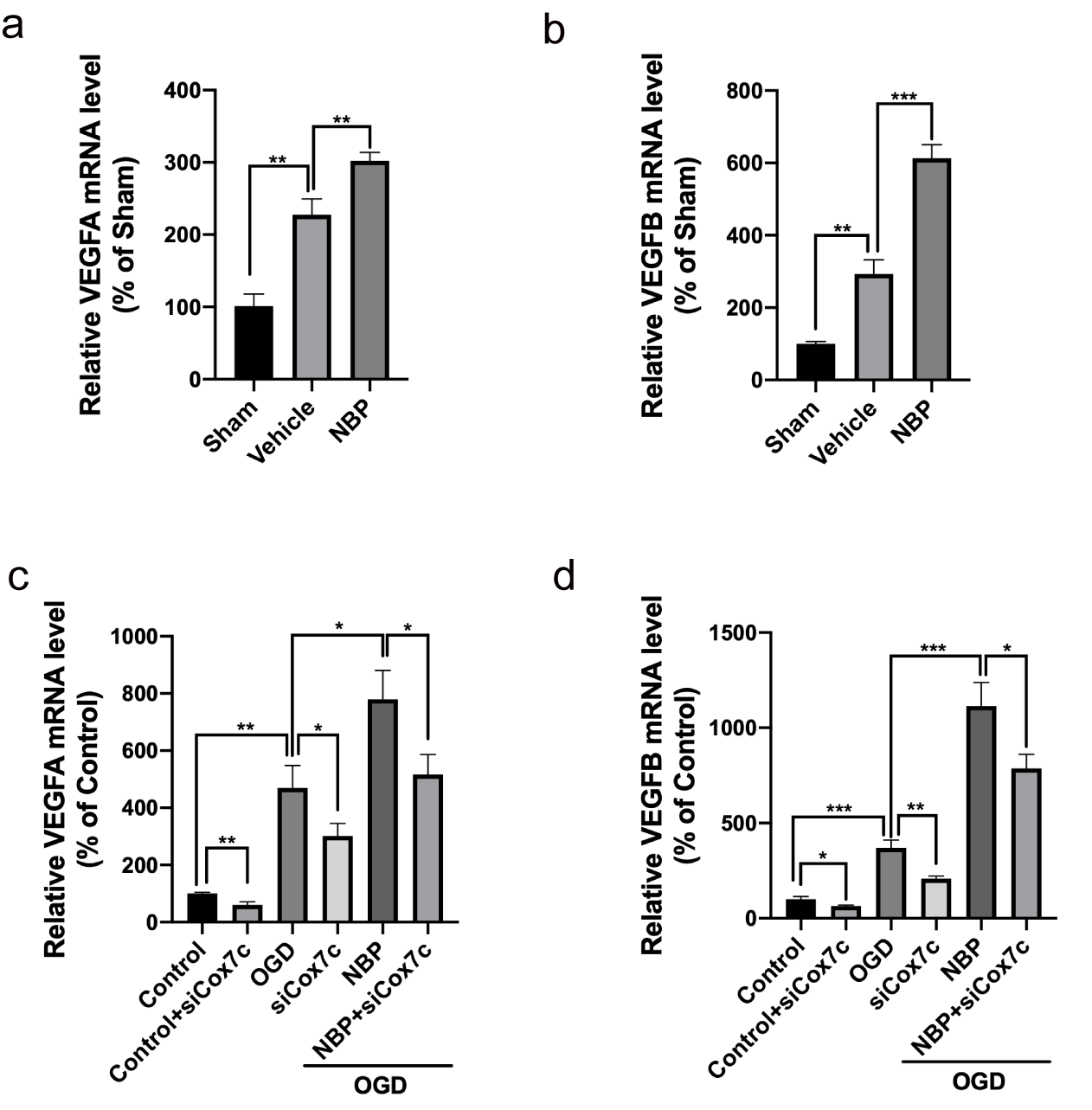

Supplement: Supplementary file 1 [file Image6.TIF]

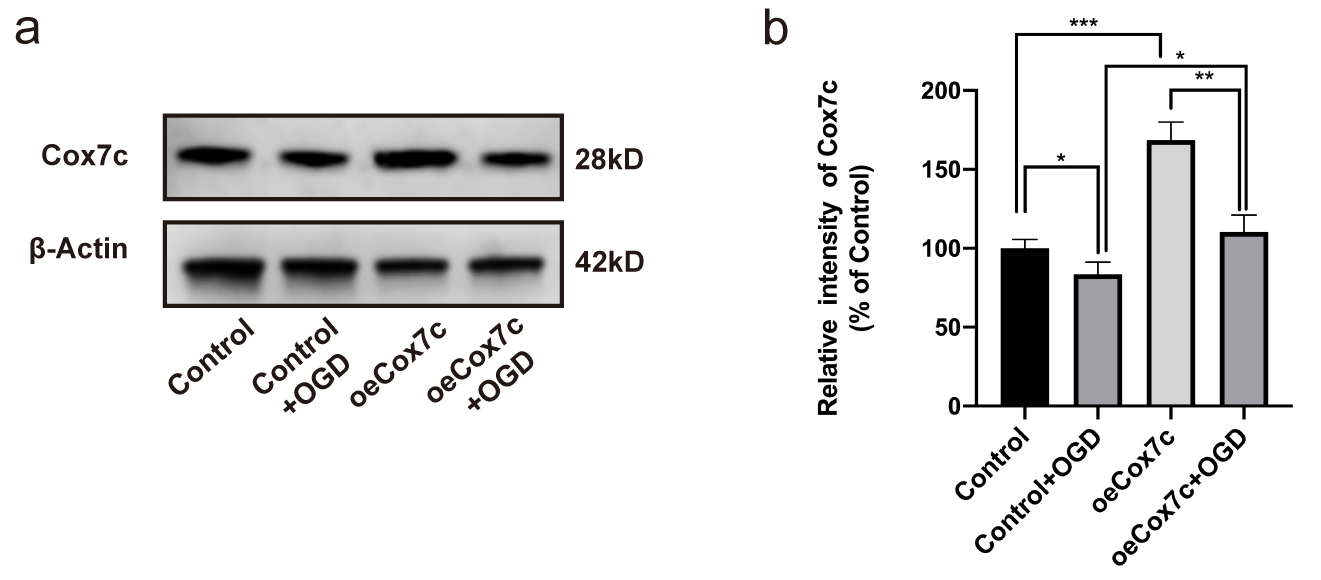

Supplement: Supplementary file 2 [file Image3.TIF]

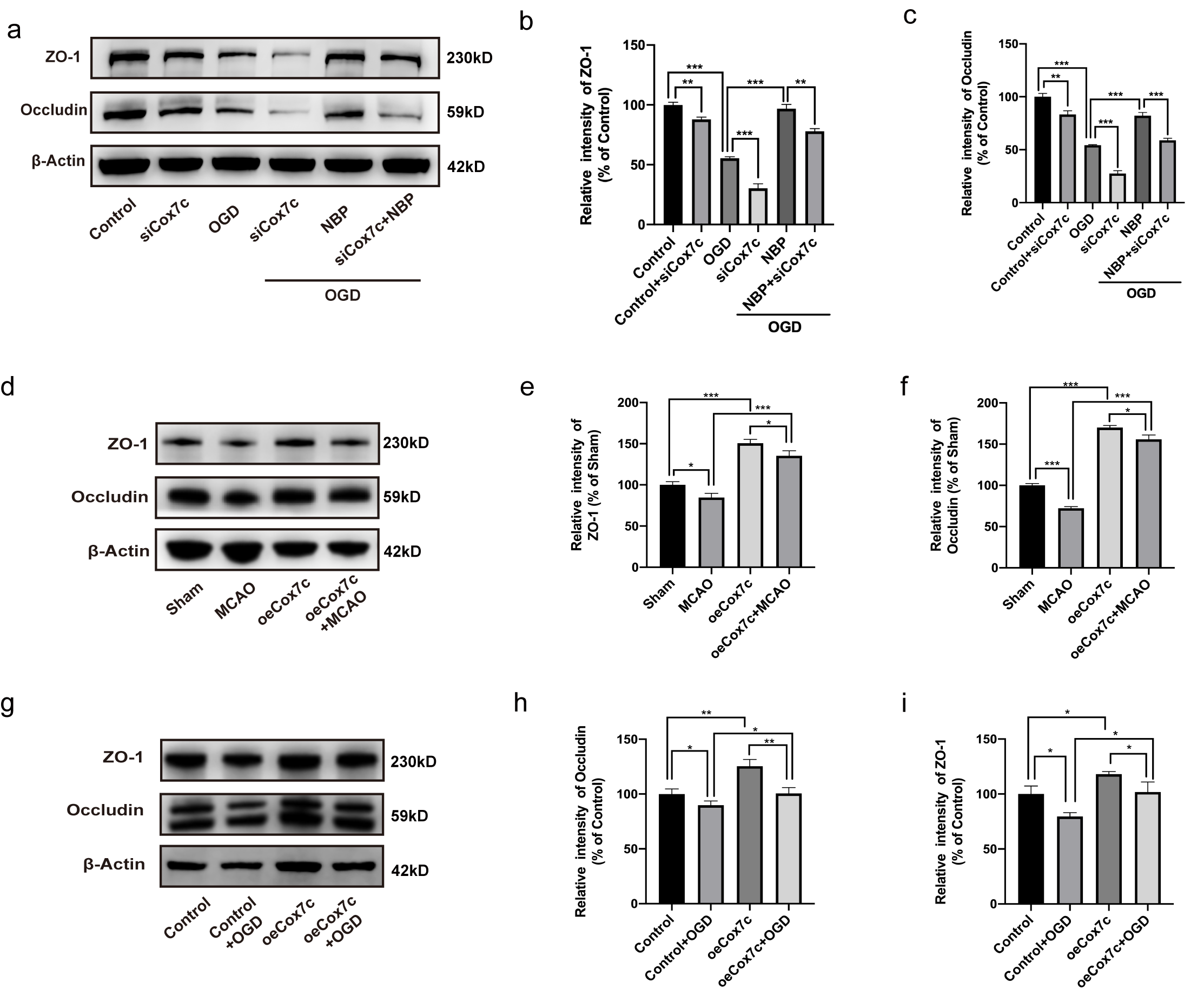

Supplement: Supplementary file 3 [file Image4.TIF]

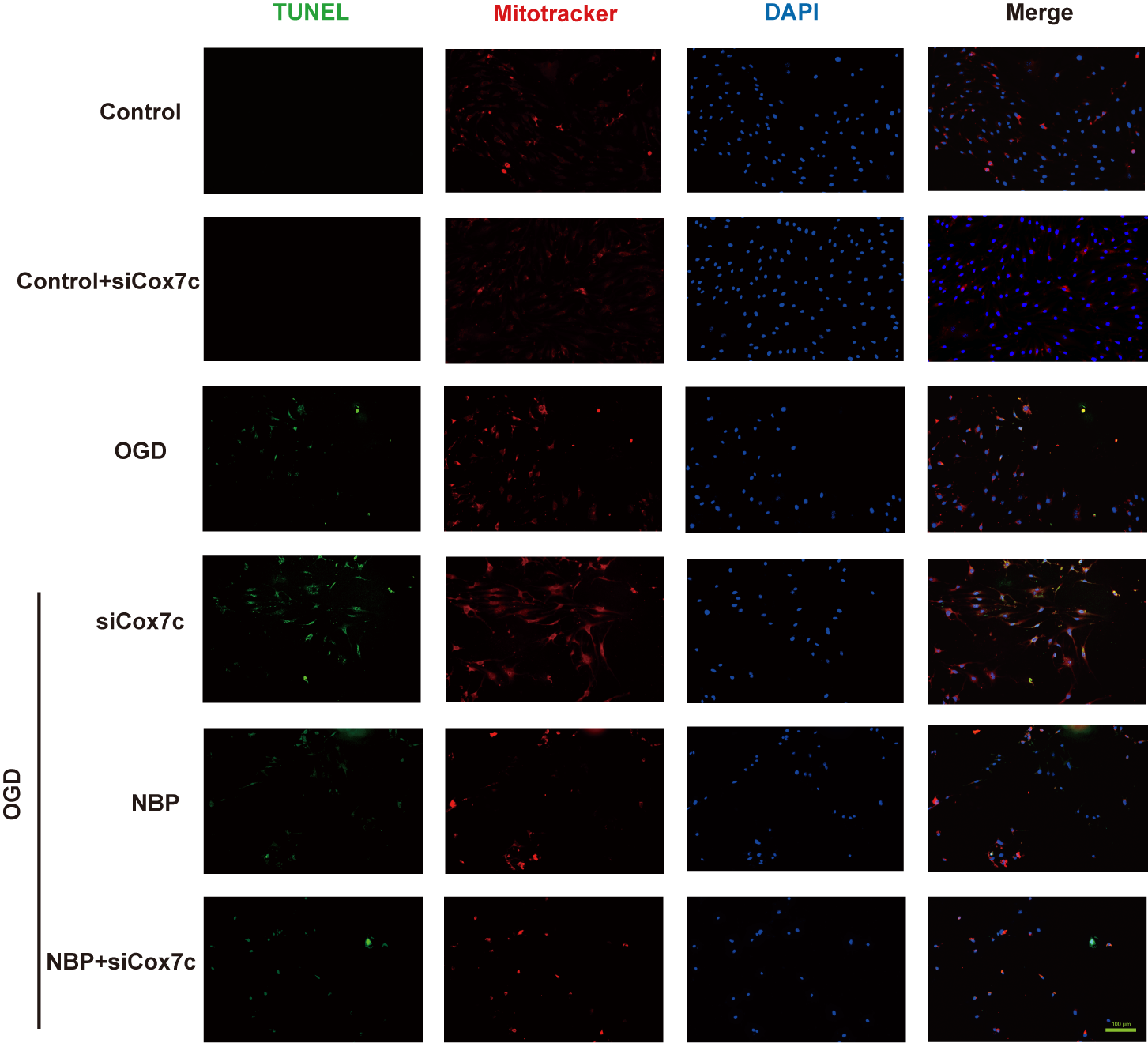

Supplement: Supplementary file 4 [file Image2.TIF]

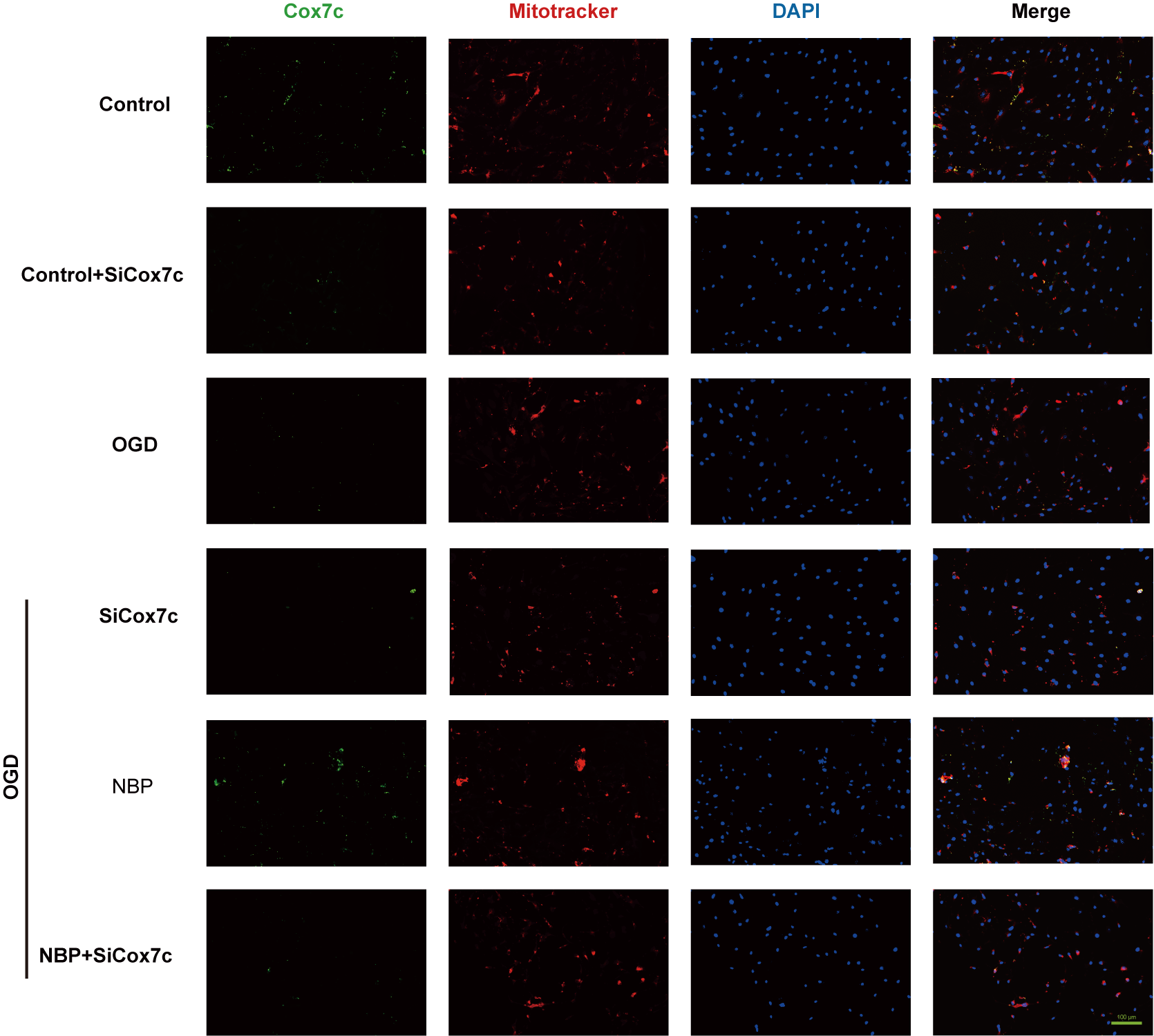

Supplement: Supplementary file 5 [file Image1.TIF]

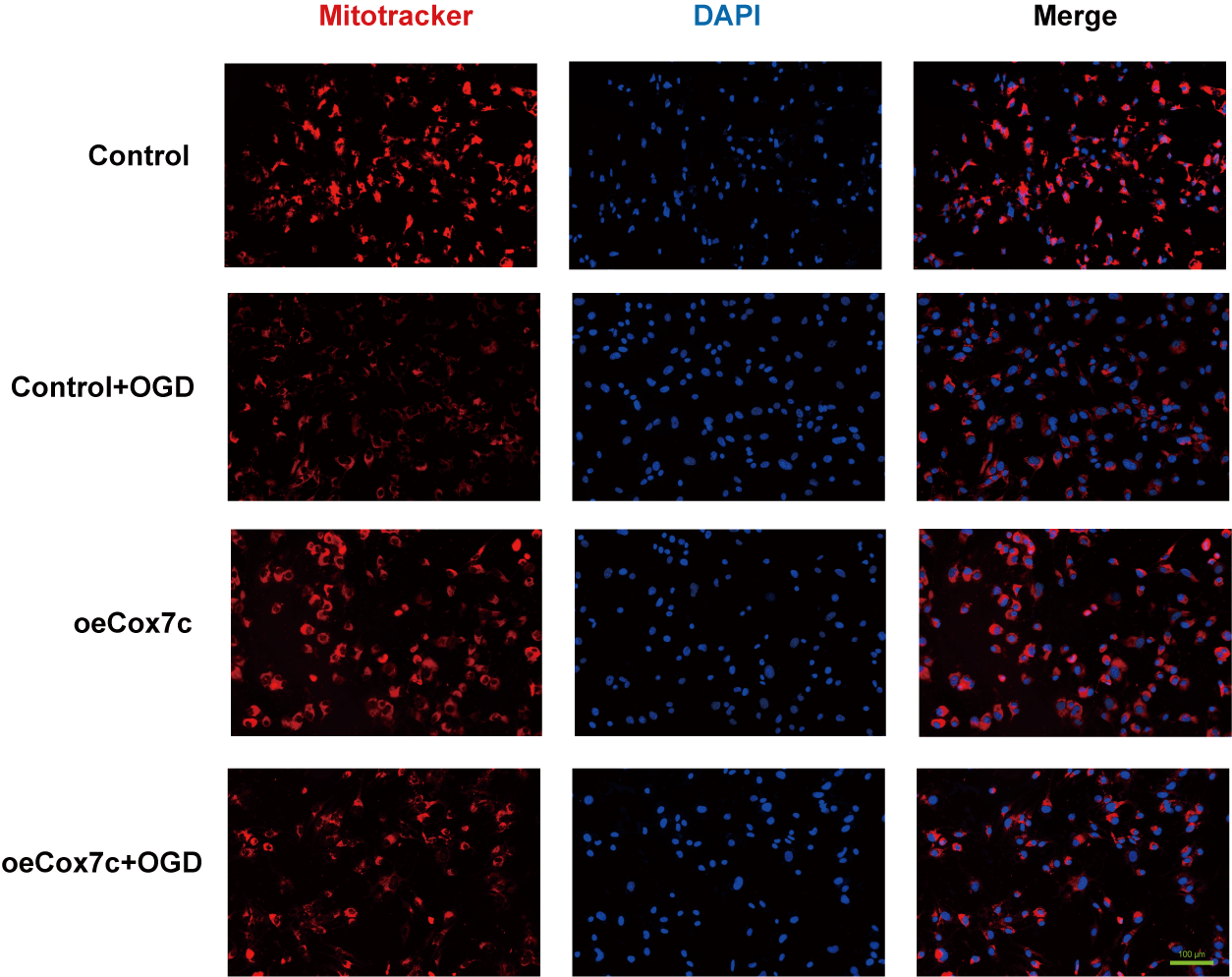

Supplement: Supplementary file 6 [file Image5.TIF]
