# Supplementary material for: Effect of Dl-3-n-butylphthalide on mitochondrial Cox7c in models of cerebral ischemia/reperfusion injury
Source: Front Pharmacol. 2023 Feb 22;14:1084564. doi: 10.3389/fphar.2023.1084564 (PMC9992206; doi:10.3389/fphar.2023.1084564)
Supplement: Supplementary file 7 [file DataSheet1.docx]

**Supplementary Figures**

**Fig. S1. Expression of Cox7c on the mitochondria of bEnd.3 cells.** Fluorescence microscopy showing Cox7c (green fluorescence) expression in OGD/R endothelial cells and co-localisation with mitochondria (red fluorescence). The data are representative of three independent experiments. Scale bars :100 μm.

**Fig.S2. Effects of NBP and siCox7c on mitochondrial apoptosis in vitro.** Fluorescence microscopy shows the expression of TUNEL (green fluorescence) and MitoTracker (red fluorescence) in OGD/R induced bEnd.3 cells.

**Fig. S3. The effects of oeCox7c on ischemia/reperfusion models in vitro.** (**A** and **B**) Effects of oeCox7c on Cox7c expression in OGD/R-induced bEnd.3 cells, and results of semi-quantitative analysis of protein grey scale. The data are representative of three independent experiments. All data are expressed as mean ± SEM. *P< 0.05, **P<0.01, ***P<0.001.

**Fig. S4.The effects of oeCox7c on the protein expression levels of ZO-1 and occludin in ischemia/reperfusion models in vivo and in vitro.** (**A** to **C**) Effects of NBP and siCox7c on the protein expression levels of ZO-1 and occludin in OGD/R induced bEnd.3 cells, as well as the results of semi-quantitative analysis of protein grey.(**D** to **F**) Effects of oeCox7c on the protein expression of ZO-1 and occludin in ischemia/reperfusion brain tissue and results of semi-quantitative analysis of protein grey scale. (**G** to **I**) Effects of oeCox7c on the protein expression levels of ZO-1 and occludin in OGD/R induced bEnd.3 cells, as well as the results of semi-quantitative analysis of protein grey. The data are representative of three independent experiments. All data are expressed as mean ± SEM. *P< 0.05, **P<0.01, ***P<0.001.

**Fig. S5. Effects of oeCox7c on mitochondrial function in vitro.** Fluorescence microscopy shows the expression of mitochondria (red fluorescence) in OGD-induced bEnd.3 cells to assess the effect of oeCox7c on mitochondria. The data are representative of three independent experiments. All data are expressed as mean ± SEM. *P< 0.05, **P<0.01, ***P<0.001. Scale bars, 100 μm.

**Fig. S6.Effects of NBP on mRNA expression of VEGFA and VEGFB in vivo and in vitro.** (**A** and **B**) Effects of NBP on mRNA expression of VEGFA and VEGFB in MCAO/R mouse brains. (**C** and **D**) Effects of NBP and siCox7c on mRNA expression of VEGFA and VEGFB in OGD/R induced bEnd.3 cells. The data are representative of three independent experiments. All data are expressed as mean ± SEM. *P< 0.05, **P<0.01, ***P<0.001.
